# Evaluation of Safety of Treatment With Anti–Epidermal Growth Factor Receptor Antibody Drug Conjugate MRG003 in Patients With Advanced Solid Tumors: A Phase 1 Nonrandomized Clinical Trial

**DOI:** 10.1001/jamaoncol.2022.0503

**Published:** 2022-05-05

**Authors:** Miao-Zhen Qiu, Yang Zhang, Ye Guo, Wei Guo, Weiqi Nian, Wangjun Liao, Zhongyuan Xu, Wenxue Zhang, Hong-Yun Zhao, Xiaoli Wei, Liqiong Xue, Wenbo Tang, Yunteng Wu, Guoxin Ren, Ling Wang, Jingle Xi, Yongshuai Jin, Hu Li, Chaohong Hu, Rui-Hua Xu

**Affiliations:** 1 Department of Medical Oncology, Sun Yat-sen University Cancer Center, State Key Laboratory of Oncology in South China, Collaborative Innovation Center for Cancer Medicine, Sun Yat-sen University, Guangzhou, People’s Republic of China; 2 Department of Clinical Research, State Key Laboratory of Oncology in South China, Collaborative Innovation Center for Cancer Medicine, Sun Yat-sen University Cancer Center, Guangzhou, Guangdong, China; 3 Department of Oncology, Shanghai East Hospital Tongji University School of Medicine, Shanghai, China; 4 Ninth People's Hospital, College of Stomatology, Shanghai Jiao Tong University School of Medicine, Shanghai, China; 5 Chongqing Cancer Institute, Chongqing University Cancer Hospital, Chongqing, China; 6 Department of Oncology, Nanfang Hospital, Southern Medical University, Guangzhou, China; 7 Radiation Oncology Department, Tianjin Medical University General Hospital, Tianjin, Tianjin, China; 8 Lepu Biopharma Co, Beijing, China; 9 Shanghai Miracogen Inc, Shanghai, China; 10 Research Unit of Precision Diagnosis and Treatment for Gastrointestinal Cancer, Chinese Academy of Medical Sciences, Guangzhou, People’s Republic of China

## Abstract

**Importance:**

The antibody drug conjugate drug MRG003 comprises an anti–epidermal growth factor receptor (EGFR) humanized immunoglobulin G1 monoclonal antibody that is conjugated with monomethyl auristatin E via a valine-citrulline linker. There is currently insufficient evidence of this drug's safety and efficacy.

**Objective:**

To evaluate the safety and maximum tolerated dose of MRG003 in a phase 1a study and investigate the preliminary antitumor activity in EGFR-expressing patients in a phase 1b study.

**Design, Setting, and Participants:**

This nonrandomized open-label, single-arm, phase 1, multicenter study of solid tumors was divided into 2 parts, phase 1a dose escalation and phase 1b dose expansion. Patients with advanced or metastatic solid tumors who had failed outcomes from or were not able to receive standard treatment were enrolled in phase 1a without EGFR prescreening. Phase 1b recruited EGFR-positive patients with refractory advanced squamous cell carcinomas of the head and neck (SCCHN), nasopharyngeal carcinoma (NPC), and colorectal cancer (CRC). This study was conducted at 7 Chinese centers between April 11, 2018, and March 29, 2021 (data cutoff date). Data analysis took place between April 2021 and June 2021.

**Interventions:**

An intravenous dose of 0.1 to 2.5 mg/kg of MRG003 was administered every 3 weeks during phase 1a. During phase 1b, patients were administered the recommended dose identified in phase 1a.

**Main Outcomes and Measures:**

The primary end points were dose-limiting toxic effects in phase 1a and objective response rate in phase 1b. The safety, tolerability, immunogenicity, and pharmacokinetics of MRG003 were assessed. Tumor assessment was evaluated by RECIST 1.1.

**Results:**

Twenty-two patients (mean [range] age, 54.5 [32.0-67.0] years; 9 women [41%]) were enrolled in phase 1a and 39 patients (mean [range] age, 50.4 [27.0-75.0] years; 8 women [21%]) in phase 1b. The recommended dose was identified as 2.5 mg/kg. Eighty-nine percent of adverse events (AEs) were associated with MRG003 treatment, and most AEs were grade 1 to 2. Nineteen patients (31%) reported grade 3 or greater treatment-related AEs, including hyponatremia, leukocytopenia, neutropenia, increased aspartate aminotransferase levels, and febrile neutropenia. In phase 1a, 1 patient (5%) achieved a partial response, and 5 (23%) achieved stable disease. In phase 1b, 8 patients (21%) achieved a confirmed partial response, and 12 (31%) achieved stable disease. The objective response rates for SCCHN, NPC, and CRC were 40%, 44%, and 0%, and the disease control rates were 100%, 89%, and 25%, respectively.

**Conclusions and Relevance:**

The findings of this nonrandomized clinical trial suggest that MRG003 showed a manageable safety profile and promising antitumor activity in patients with EGFR-positive NPC and SCCHN.

**Trial Registration:**

Clinicaltrials.gov Identifier: NCT04868344

## Key Points

**Question:**

What is the safety and antitumor activity of MRG003 in patients with advanced solid tumors?

**Findings:**

In this phase 1 clinical trial of 61 patients with advanced or metastatic solid tumors, treatment with MRG003 exhibited manageable safety and showed encouraging antitumor activity in squamous cell carcinomas of the head and neck and nasopharyngeal carcinoma, with a confirmed objective response rates of 40% and 44%, respectively.

**Meaning:**

The study findings suggest the safety of treatment with MRG003 and an acceptable tolerance in most patients with epidermal growth factor receptor–expressing solid tumors, as well as encouraging antitumor activity in patients with squamous cell carcinomas of the head and neck and nasopharyngeal carcinoma.

## Teaser

This nonrandomized clinical trial examines the safety and maximum tolerated dose of MRG003 in a phase 1a study of patients with solid tumors and investigates the preliminary antitumor activity in epidermal growth factor receptor–expressing patients in a phase 1b study.

## Introduction

Epidermal growth factor receptor (EGFR) is 1 of the 4 members of the *ErbB* family of tyrosine kinase receptors,^1^ and it plays a critical role in the modulation of cell proliferation, differentiation, migration, survival, and adhesion. Its overexpression or variance may contribute to the development of tumors.^2,3,4^ Epidermal growth factor receptor is reported to be overexpressed in various solid tumors, including squamous cell carcinomas of the head and neck (SCCHN), nasopharyngeal carcinoma (NPC), and colorectal cancer (CRC).^5,6,7^

Antibody drug conjugates (ADCs) are an emerging class of cancer therapeutics that combines several mechanisms of action to improve efficacy and reduce the frequency and severity of adverse effects.^8^ The drug MRG003 is a novel ADC molecule of an anti-EGFR humanized immunoglobulin (Ig) G1 monoclonal antibody (mAb) conjugated with monomethyl auristatin E via a valine-citrulline linker. The potent antitumor activity of MRG003 has been demonstrated in preclinical xenograft mouse models. In this article, we report the results of what is to our knowledge the first dose escalation and dose-expansion study of MRG003 to involve human participants in patients with solid tumors.

## Methods

### Patients

Phase 1a recruited refractory patients with advanced or metastatic solid tumors who had progressed beyond known lines of therapy without EGFR prescreening. Phase 1b recruited EGFR-positive patients with refractory advanced SCCHN, NPC, and CRC.

This trial was approved by the institutional ethics committee review board at each site, and conducted in accordance with Good Clinical Practice guidelines and the Declaration of Helsinki. All the patients signed informed consent. The trial protocol is described in Supplement 1.

### Treatment Design

During phase 1a, the first 2 low-dose cohorts followed a modified accelerated titration dose escalation design, and the subsequent 6 dose cohorts followed a conventional 3 + 3 dose escalation design. The initial dose of MRG003 was 0.1 mg/kg, followed by 0.3, 0.6, 1.0, 1.5, 2.0, and 2.5 mg/kg. Patients were administered treatment with MRG003 every 3 weeks for a maximum of 8 cycles during both phases.

### Safety Assessment

The study evaluated adverse events (AEs) and serious AEs (SAEs) according to the National Cancer Institute Common Terminology Criteria for Adverse Events, version 4.03. A radiography assessment was performed a mean (SD) of every 6 (1) weeks, and the tumor response was evaluated according to the Response Evaluation Criteria in Solid Tumors, version 1.1.^9^

### Statistical Analysis

For safety analysis sets, patients who received at least 1 dose of MRG003 were included. Progression-free survival (PFS), overall survival (OS), and duration of response (DOR) were estimated using the Kaplan-Meier method. All analyses were performed using SAS, version 9.4 (SAS Institute).

## Results

### Patient Demographic Characteristics

Sixty-one patients received MRG003 (22 [36%] in phase 1a and 39 [64%] in phase 1b). Phase 1a comprised 2 patients with SCCHN, 3 with NPC, 15 with CRC, 1 with esophageal cancer, and 1 with duodenal cancer. Phase 1b included 13 patients with SCCHN, 14 with NPC, and 12 with CRC. The baseline characteristics are shown in Table 1.

**Table 1.** Patient Baseline Characteristics

Characteristic	Phase 1a (n = 22)	Phase 1b (n = 39)
Age, mean (range), y	54.5 (32-67)	50.4 (27-75)
Sex, No. (%)		
Female	9 (41)	8 (21)
Male	13 (59)	31(79)
BMI	22.28	22.38
ECOG PS, No. (%)		
0	10 (45)	10 (26)
1	12 (55)	29 (74)
Primary tumor, No.		
SCCHN	2	13
NPC	3	14
CRC	15	12
Other^a^	2	0
No. of prior systemic therapies, median (range)	3 (1-8)	2 (1-6)
Smoking, No. (%)		
Yes	11 (50)	13 (33)
No	11 (50)	26 (67)
Alcohol use, No. (%)		
Yes	1 (5)	3 (8)
No	21 (95)	36 (92)
EGFR status, No. (%)^b^		
0	9 (41)	0
1+^c^	4 (18)	1 (3)
2+^d^	1 (5)	12 (31)
3+^e^	2 (9)	8 (21)
4+^f^	2 (9)	18 (46)
Missing	4 (18)	0

Abbreviations: BMI, body mass index (calculated as weight in kilograms divided by height in meters squared); CRC, colorectal cancer; ECOG PS, Eastern Cooperative Oncology Group scale of performance status; EGFR, epidermal growth factor receptor; NPC, nasopharyngeal carcinoma; SCCHN, squamous cell carcinoma of head and neck.

^a^ Included 1 patient with esophageal squamous cell cancer and 1 with duodenal adenocarcinoma.

^b^ EGFR status was defined as follows: 0, no membrane staining observed; missing, no tumor tissue was detected.

^c^ Membrane staining observed in 1% to 25% of the tumor cells.

^d^ Membrane staining observed in 26% to 50% of the tumor cells.

^e^ Membrane staining observed in 51% to 75% of the tumor cells.

^f^ Membrane staining observed in 75% or greater of the tumor cells.

### Dose Escalation

No dose-limiting toxic effects were observed in the 0.1 to 2.0 mg/kg dose cohorts within 21 days after receipt of the first dose of MRG003, whereas a single dose-limiting toxic effect event was documented in the 2.5 mg/kg dose cohort (febrile neutropenia and bacterial pneumonia). Participants in phase 1b received a dose of 2.5 mg/kg. Pharmacokinetics of MRG003 are shown in eTable 1 and eFigure 1 in Supplement 2.

### Safety

Eighty-nine percent of AEs were associated with MRG003 treatment, and most AEs were grade 1 to 2. The most frequently reported AEs were rash and increased aspartate aminotransferase levels, comprising 39% each (Table 2). In phases 1a and 1b, 19 patients (31%) had grade 3 or greater treatment-related AEs, of which hyponatremia (8%), leukopenia (7%), and neutropenia (5%) were the most common (eTable 2 in Supplement 2).

**Table 2.** TRAEs in 10% or More of All Patients

TRAE	No. (%)
	All grades (n = 61)
Aspartate aminotransferase levels increased	24 (39)
Rash	24 (39)
Alopecia	20 (33)
Decreased appetite	19 (31)
Alanine aminotransferase levels increased	18 (30)
Pruritus	17 (28)
Leukocytopenia	15 (25)
Myalgia	14 (23)
Pyrexia	13 (21)
Neutropenia	12 (20)
Asthenia	12 (20)
Anemia	11 (18)
Hypoesthesia	10 (16)
Oral ulcer	9 (15)
Fatigue	8 (13)
Diarrhea	8 (13)
Weight decreased	7 (12)
Hemoglobin levels decreased	7 (12)
Pain in extremity	6 (10)
Vomiting	6 (10)
Constipation	6 (10)
Nausea	6 (10)

Abbreviation: TRAE, treatment-related adverse event.

In phase 1a, 1 patient experienced a MRG003-related SAE of grade 4 febrile neutropenia that was followed by grade 5 lung infection. In phase 1b, 1 patient experienced a MRG003-related SAE of grade 4 febrile neutropenia that was followed by sepsis and hyperglycemic hyperosmolar nonketotic syndrome; the patient eventually died of multiorgan failure.

### Antitumor Activity

In phase 1a, 22 patients received at least 1 dose of MRG003. One patient achieved partial response (PR), 5 achieved stable disease (SD), and 13 had progressive disease (PD) (Figure, A). The objective response rate (ORR) and disease control rate (DCR) were 4.5% (1 of 22) and 27.3% (6 of 22), respectively. All PR and SD were observed in EGFR-positive patients. The ORR and DCR in the EGFR-positive subgroup were 11.1% and 66.7%, respectively.

**Figure.**
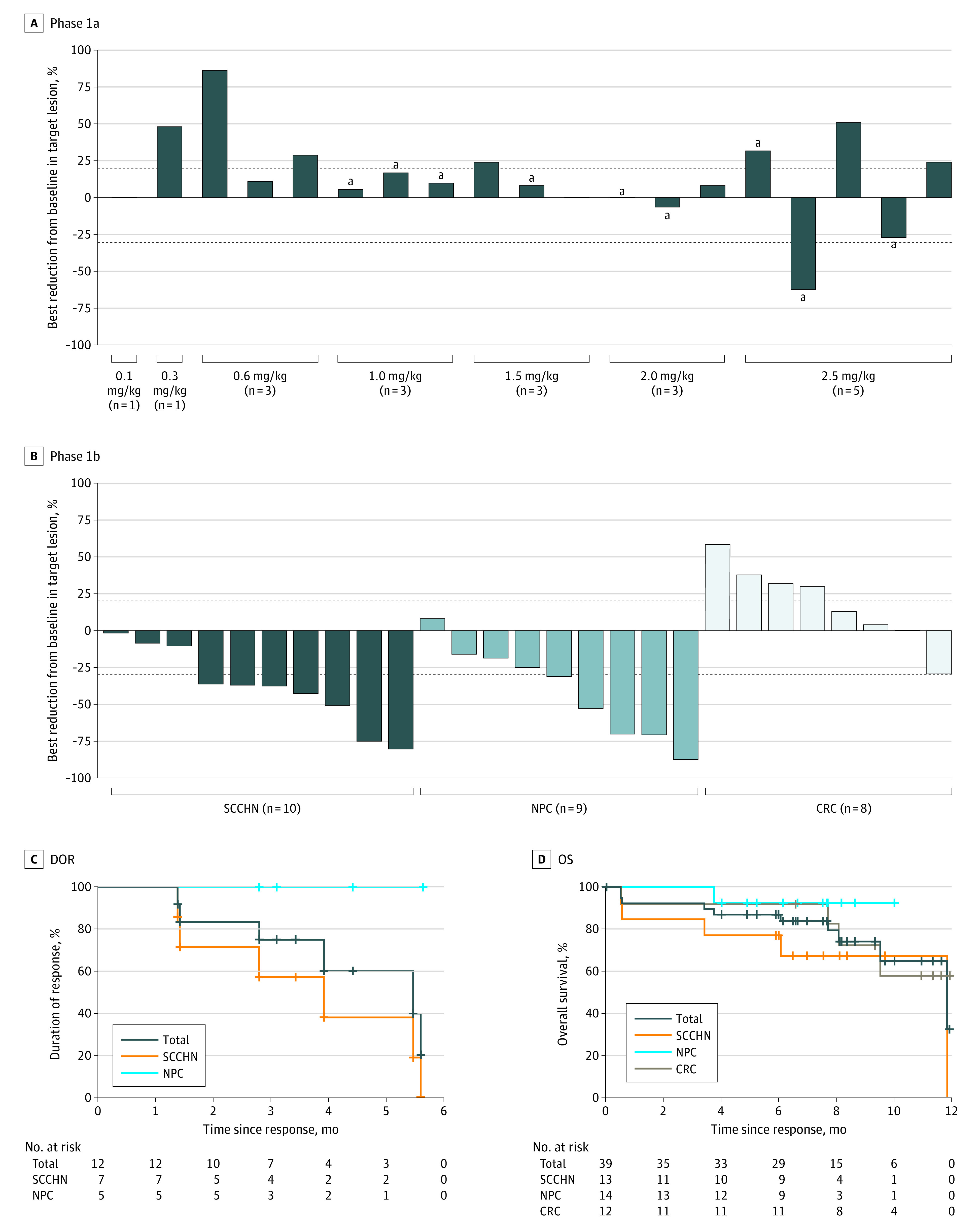
Safety for Patients in Phases 1a Dose Escalation and 1b Dose Expansion A and B, Most substantial reductions of tumor size in phases 1a and 1b. C and D, Kaplan-Meier curves of duration of response (DOR) and overall survival (OS) in all phase 1b patients (n = 39), including squamous cell carcinoma of the head and neck (SCCHN; n = 13), nasopharyngeal cancer (NPC; n = 14), and colorectal cancer (CRC; n = 12). The dashed line above represented a 20% increase and the dashed line below represented a 30% decrease. ^a^Epidermal growth factor receptor and immunohistochemistry positive.

In phase 1b, all 39 patients were EGFR positive, and the response evaluation was available for 27 patients (69%). Eight patients achieved confirmed PR, 12 achieved SD, and 7 experienced PD (Figure, B). The ORR and DCR were 20.5% (8 of 39) and 51.3% (20 of 39), respectively. The ORR for SCCHN, NPC, and CRC was 40%, 44%, and 0%, and the DCR for SCCHN, NPC, and CRC were 100%, 89%, and 25%, respectively.

The DOR, OS, and PFS were analyzed in patients during phase 1b. The median DOR of all patients was 5.6 months (SCCHN: DOR, 5.6 months; 95% CI, 2.8-5.6; NPC: not estimable) (Figure, C) as of the data cutoff date. The median PFS of all patients was 2.8 months (95% CI, 1.2-4.1 months), and the PFS of SCCHN, NPC, and CRC was 2.8 (95% CI, 0.6-6.8) months, 4.0 (95% CI, 1.2-not reached) months, and 1.2 (95% CI, 0.5-2.8) months, respectively (eFigure 2 in Supplement 2). Only the SCCHN cohort reached the median OS as of the data cutoff date, which was 11.8 (95% CI, 3.4-11.8) months (Figure, D). There was a positive association between EGFR expression and outcomes of treatment with MRG003 (eTable 3 in Supplement 2).

## Discussion

Compared with US Food and Drug Administration–approved EGFR-targeting mAbs, including cetuximab and panitumumab, rash, pruritus, and other skin toxic effects were less frequently observed during treatment with MRG003 than what has been reported for cetuximab or panitumumab.^10^ Two deaths were associated with febrile neutropenia, subsequent infections, and a cascade of events, which were most likely associated with monomethyl auristatin E, suggesting that the hematological toxic effects of MRG003 should be monitored closely and controlled in clinical practice.

Immune checkpoint inhibitors are the second-line therapy for metastatic NPC or SCCHN, with an ORR of 10% to 26%.^11,12,13,14,15^ The antitumor activity of MRG003 in patients NPC and SCCHN who underwent substantial pretreatment looked promising in this study but require a larger study sample to be further confirmed. Moreover, in phase 1b, 15 patients (38%) had previously received anti-EGFR mAb therapy (cetuximab or nituzumab), including 3 patients with CRC, 6 with SCCHN, and 6 with NPC. After treatment with MRG003, the ORR for SCCHN, NPC, and CRC was 50%, 50%, and 0. The DCR for SCCHN, NPC, and CRC was 83%, 67%, and 33%, respectively. Therefore, even for patients who did not experience positive outcomes after receiving prior therapy that contained an anti-EGFR mAb, treatment with MRG003 still showed some antitumor activity in patients with SCCHN and NPC. The outcomes of treatment with MRG003 for CRC were not substantial enough to warrant further studies.

### Limitations

This study is limited by the small sample size. The promising antitumor outcome needs to be further confirmed in a cohort with more patients.

## Conclusions

In this nonrandomized clinical trial, MRG003 administered at a dose of 2.5 mg/kg appeared to be tolerable in most of the treated patients with advanced or metastatic solid tumors, but the optimal dosing scheme, as well as the risk and benefit ratio of MRG003, requires further investigation. The results suggest that its antitumor activity in EGFR-expressing patients with advanced SCCHN and NPC is encouraging. Phase 2 studies in patients with advanced SCCHN and NPC have been initiated.

## Notes

Supplement 1. Trial protocol

Supplement 2. eTable 1. Pharmacokinetics for phase Ia dose-escalation and phase Ib dose-expansion eTable 2. Treatment-related AEs (TRAEs) ≥ Grade 3 of all patients eTable 3. The relationships between EGFR status, BOR, PFS, and OS eFigure 1. Pharmacokinetics of MRG003 following the first administration eFigure 2. Progression-free survival for phases Ib dose-expansion

Supplement 3. Data sharing statement
