# Supplementary material for: Evaluation of Safety of Treatment With Anti–Epidermal Growth Factor Receptor Antibody Drug Conjugate MRG003 in Patients With Advanced Solid Tumors: A Phase 1 Nonrandomized Clinical Trial
Source: JAMA Oncol. 2022 May 5;8(7):1042–6. doi: 10.1001/jamaoncol.2022.0503 (PMC9073657; doi:10.1001/jamaoncol.2022.0503)
Supplement: Supplement 1. — Trial protocol [file jamaoncol-e220503-s001.pdf]

First-in-human dose escalation and dose-expansion study of anti-EGFR antibody drug conjugate MRG003 in patients with advanced solid tumors

Protocol number: MRG003-001

Principal investigator: Ruihua Xu

Sponsor: Shanghai Miracogen Inc.

Version NO.: 5.0

02 December 2019

Signature Page

INVESTIGATOR/SPONSOR STATEMENT:

I have read this protocol and agree to conduct the study in accordance with all aspects of the protocol. I will provide copies of this protocol to the staff participating in the clinical trial and use the information provided by the sponsor under my supervision. I agree to conduct this study in compliance with all applicable regulations and China's Good Clinical Practice (GCP).

Sponsor: Sun Yat-sen University Cancer Center

Principal Investigator (Signature):

Date: MM/DD/YY

Sponsor: SHANGHAI MIRACOGEN INC.

Project leader of sponsor (signature):

Date: MM/DD/YY

CONTENTS

1 LIST OF ABBREVIATIONS **9**

2 PROTOCOL SYNOPSIS **12**

3 KEY INVESTIGATOR OR STUDY SITE **21**

3.1 Sponsor Responsible 21

3.2 Principal Investigator 21

3.3 Safety Monitoring Committee (SMC) 21

3.4 Central Laboratory 21

4 INTRODUCTION: STUDY BACKGROUND AND BASIS **23**

4.1 Background information 23

4.1.1 Solid tumor background *23*

4.1.2 Background of relevant therapeutic drug *23*

4.2 Basis for Study 24

4.2.1 Limitations of available treatments *24*

4.2.2 Study drug *26*

4.2.2.1 Drug name: MRG003 for Injection 26

4.2.2.2 Major metabolic pathways 27

5 TRIAL OBJECTIVE **30**

5.1 Primary Trial Objectives 30

5.2 Secondary Trial Objectives 30

5.3 Explore trial objectives 30

6 TRIAL DESIGN AND OBSERVATION INDICATORS **31**

6.1 Total trial design 31

6.2 Observation indicators 32

6.2.1 Primary Outcome Measures *32*

6.2.2 Secondary outcome measures *32*

6.2.3 Exploratory Outcome Measures *33*

6.3 Drug tolerability assessment 33

6.3.1 Selection of starting dose *33*

6.3.2 Selection of maximum dose *34*

6.3.3 Dose Escalation Scheme *34*

6.3.4 Dose-limiting toxicity, maximum tolerated dose and recommended phase II dose *35*

6.3.4.1 Judgment criteria for dose limiting toxicity (DLT) 35

6.3.4.2 Determine MTD or RP2D 37

6.3.5 SMC Responsibilities *37*

6.4 Evaluation of Kinetics 37

6.5 Efficacy Assessments 38

7 TRIAL, WITHDRAWAL, DISCONTINUATION 38

7.1 Number of subjects 38

7.2 Study site 39

7.3 Inclusion criteria 39

7.4 Exclusion Criteria 40

7.5 Subject Withdrawal Criteria 42

7.5.1 Investigator's decision to withdraw a subject *42*

7.5.2 Withdrawal by subject *43*

7.6 Criteria for Discontinuation 43

7.7 Exclusion criteria 43

7.8 Criteria for Drop-out Cases 44

8 STUDY DRUG 45

8.1 Information about the study drug 45

8.1.1 Study Drug Shipment and Transfer *45*

8.1.2 Study Drug Ingredients, Appearance, Packaging, and Labeling *45*

8.1.3 Study Drug Storage and Stability Testing *45*

8.1.4 Study Drug Preparation *45*

8.1.5 Dose and Schedule *46*

8.1.6 Administration method and administration period *46*

8.2 Dose Modifications, Modifications, and Delays 46

8.3 Management and Responsibilities for Study Drug 47

8.4 Return and/or Destruction of Study Drug 48

8.4.1 Partially used or unused study drug *48*

8.4.2 Possible recall *48*

9 TRIAL PROCEDURES/VISIT SCHEDULE 49

9.1 Visit Schedule 49

9.2 Prescreening (applicable for Phase Ib) 55

9.3 Screening Period (Day -28 to Day -1) 55

9.4 First dosing period (Day -1 ~ 1) 56

9.4.1 Admission to Phase I Clinical Trials Unit *56*

9.4.2 Before intravenous drip of MRG003 ..... *57*

9.4.3 First IV drip (Day 1) *57*

9.5 Ia Treatment Period and Post-Treatment Assessments 60

9.6 Ib Treatment Period and Post-Treatment Assessments 61

9.7 Efficacy and Survival Follow-up 62

9.8 Early termination of treatment 62

10 TEST OPERATION GUIDELINE 64

10.1 General operation 64

10.2 Laboratory assessments 64

10.2.1 Clinical Laboratory Assessments *64*

10.2.1.1 Blood routine examination 64

10.2.1.2 Coagulation test 64

10.2.1.3 Blood biochemistry 64

10.2.1.4 Carcinoembryonic antigen test 65

10.2.1.5 Urine test 65

10.2.1.6 Serum pregnancy test 65

10.2.1.7 Infectious Disease Testing 65

10.2.2 Special Experimental Assessments *65*

10.2.2.1 Genetic testing 65

10.2.2.2 Determination of metabolic parameters of study drug 66

10.2.2.3 Study Drug Immunogenicity Assay 66

10.3 Permitted concomitant medications and treatments 66

10.4 Prohibited concomitant medications and treatments 67

10.5 Post-Study Treatment 67

10.6 Treatment Accountability and Compliance 67
11 SAFETY ASSESSMENTS 69
11.1 Definition of different types of adverse events 69
11.1.1 Adverse Event Definitions 69
11.1.2 Definition of Serious Adverse Event 69
11.2 Classification of adverse events 70
11.2.1 Severity of adverse event 70
11.2.2 Relationship of adverse event to study drug 70
11.3 Frequency and duration of adverse events 72
11.4 Adverse event monitoring 72
11.5 Recording and reporting of adverse events 72
11.5.1 AE recording 72
11.5.2 SAE Reporting 72
11.5.3 Drug-induced liver injury 73
11.5.4 Serious Adverse Events Due to Disease Progression 73
11.5.5 Death 73
11.5.6 Pregnancy 74
11.5.7 Overdose 74
12 CLINICAL MONITORING 75
12.1 Investigator Responsibilities 75
12.2 Sponsor Responsibilities 75
13 DATA MANAGEMENT 77
13.1 Requirements for original documents 77
13.2 Case Report Forms and Precautions 77
13.3 Application of Computer System 77
13.4 Data collection 77
13.5 Data Management and Quality Control 77
13.6 Data Retention 78
13.7 Modification to Test Protocol 78

13.8 PRINCIPLES FOR PUBLICATION AND SHARING OF TRIAL RESULTS 79
14 STATISTICAL ANALYSIS 80
14.1 SAMPLE SIZE 80
14.2 STATISTICAL ANALYSIS PLAN 80
14.3 DATA ANALYSIS SET 80
14.4 STATISTICAL ANALYSIS METHODS 81
14.4.1 Overall Analytical Method 81
14.4.2 Baseline Analysis Methods 81
14.4.3 Analysis method of primary outcome measures 81
14.4.3.1 Primary Outcome Measures in Part Ia 81
14.4.3.2 Primary Outcome Measures in Part Ib 81
14.4.4 Analysis method of secondary outcome measures 81
14.4.4.1 Secondary Outcome Measures in Phase Ia 82
14.4.4.2 Secondary Outcome Measures in Phase Ib 82
14.4.5 Safety Analysis Methods 83
15 QUALITY ASSESSMENT AND MANAGEMENT 84
15.1 TRIAL MONITORING 84
15.2 TRIAL AUDITS 84
15.3 STUDY INSPECTION 84
16 ETHICS RELATED 85
16.1 SIGNATURE OF INFORMED CONSENT FORM 85
16.2 PROTECT PATIENT PRIVACY 86
17 INTELLECTUAL PROPERTY 86
18 REFERENCES 87
19 APPENDICES 88
19.1 APPENDIX I: PERFORMANCE STATUS SCORING CRITERIA (ECOG) 88
19.2 APPENDIX II: NEW RESPONSE EVALUATION CRITERIA IN SOLID TUMORS: REVISED RECIST GUIDELINES (VERSION 1.1) 88
19.3 APPENDIX III: CANCER DRUG STANDARD TERMINOLOGY ADVERSE EVENT EVALUATION SYSTEM (CTCAE V4.03) 91

212 # 1 LIST OF ABBREVIATIONS

Abbreviation	English full name
ADC	Antibody Drug Conjugate
AE	Adverse Event
ADA	Anti-Drug Antibody
ADCC	Antibody Dependent Cell-mediated Cytotoxicity
ALT	Alanine Aminotransferase
ALP	Alkaline Phosphatase
AST	Aspartate Aminotransferase
AUC	Area Under Curve
BRM	Biological Response Modifiers
BSC	Best Supportive Care
CDC	Dependent Complement Cytotoxicity
CL	Clearance
Cmax	Maximum Concentration
CR	Complete Response
ECRF	Electronic Case Report Form
CRU	Clinical Research Unit
CT	Computed Tomography
CTCAE	Common Terminology Criteria for Adverse Events
CDISC	Clinical Data Interchange Standards Consortium
DLT	Dose Limiting Toxicity
DOR	Duration of Response
ECG	Electrocardiogram
ELISA	Enzyme-Linked Immunosorbent Assay
ECOG	Eastern Cooperative Oncology Group
EDC	Electronic Data Capture
EGFR	Epidermal Growth Factor Receptor
FAS	Full Analysis Set
FIH	First-in-human

Abbreviation	English full name
GCP	Good Clinical Practice
G-CSF	Granulocyte Colony Stimulating Factor
GM-CSF	Granulocyte Macrophage Colony Stimulating Factor
GLP	Good Laboratory Practice
HER	Human EGF Receptor
HNSTD	Highest Non-Toxic Dose
ICF	Informed Consent Form
ICH	International Concil on Harmonization
MAb	Monoclonal Antibody
MMAE	Monomethyl Auristatin E
MRI	Magnetic Resonance Imaging
MTD	Maximum Tolerated Dose
ORR	Objective Response Rate
OS	Overall Survival
PD	Progress Disease
PFS	Progression Free Survival
PK	Pharmacokinetics
PPS	Per Protocol Set
PR	Partial Response
Q3W	Every 3 weeks
RECIST	Response Evaluation Criteria in Solid Tumors
RP2D	Recommended Phase II Dose
SAE	Serious Adverse Event
SAS	Statistics Analysis System
SD	Stable Disease
SOP	Standard Operating Procedure
SS	Safety Set
T1/2	Half-life
TAb	Total Antibody

Abbreviation	English full name
TEAEs	Treatment Emergent Adverse Event
Tmax	Time to Reach Cmax
TKI	Tyrosine Kinase Inhibitor
UCD	Unconjugated Drug
ULN	Upper Limit of Normal
Vd	Apparent Volume of Distribution

228

2 PROTOCOL SYNOPSIS

Title	First-in-human dose escalation and dose-expansion study of anti-EGFR antibody drug conjugate MRG003 in patients with advanced solid tumors
Sponsor	Shanghai Miracogen Inc.
Clinical trial phase	Phase I (including Phase Ia dose escalation part and Phase Ib dose expansion part)
Study drug	MRG003 (Antibody Conjugated Drug, ADC) for Injection<br><br>Specification: 20 mg/vial Dosage form: Injectable lyophilized drug product
Primary study objective	The primary objectives of the Phase Ia dose-escalation study are:<br><br>• To determine the maximum tolerated dose (MTD) or recommended Phase II dose (RP2D) of MRG003<br><br>The primary objectives of the Phase Ib dose expansion phase of the study are:<br><br>• To evaluate the antitumor activity of MRG003 in patients with advanced colorectal cancer, head and neck squamous cell carcinoma and nasopharyngeal carcinoma
Secondary study objectives	Secondary objectives of the Phase Ia dose-escalation study:<br><br>• To evaluate the kinetic (PK) characteristics of the drug in patients with advanced solid tumors by kinetic study;<br><br>• To observe the safety of MRG003 in patients with advanced solid tumors;<br><br>• To assess the immunogenicity of MRG003 in patients with advanced solid tumors;<br><br>• To assess the antitumor activity of MRG003 in patients with advanced solid tumors.<br><br>Secondary objectives of the Phase Ib dose expansion phase of the study:<br><br>• To evaluate the safety and tolerability of MRG003 in patients with advanced colorectal cancer, head and neck squamous cell carcinoma and nasopharyngeal carcinoma;<br><br>• Evaluate the kinetic characteristics of MRG003;<br><br>• To evaluate the immunogenicity of MRG003 in patients with advanced colorectal cancer, head and neck squamous cell carcinoma and nasopharyngeal carcinoma.
Explore trial objectives	• By detecting the expression level of epidermal growth factor receptor (EGFR) in tumor pathological tissues of subjects, the correlation between EGFR and efficacy indicators will be explored.

Number of Sites	4-8
Indications for Use	Patients with advanced solid tumors, including advanced colorectal cancer, head and neck squamous cell carcinoma and nasopharyngeal carcinoma
Subject Population	Phase Ia: approximately 26 patients with solid tumors for dose escalation to determine MTD or RP2D;<br><br>Phase Ib: 36 evaluable patients with advanced colorectal cancer, head and neck squamous cell carcinoma and nasopharyngeal carcinoma (12 patients for each group).
Overall design	First-in-human, multicenter, non-randomized, single-arm, open-label, dose escalation, dose expansion, safety, efficacy clinical trial
Test design	This study is designed in two parts: the first part of Phase Ia dose escalation study is a single-center, non-randomized, single-arm, open-label clinical trial for the first time in human to determine the maximum tolerated dose (MTD) or recommended Phase II dose (RP2D) of MRG003 in patients with solid tumors; the second part of Phase Ib dose expansion study is a multi-center, non-randomized, single-arm, open-label clinical trial to preliminarily assess the efficacy of MRG003 for advanced colorectal cancer, head and neck squamous cell carcinoma and nasopharyngeal carcinoma. Both parts will evaluate the safety, tolerability, immunogenicity and kinetics of MRG003 in human.<br><br>In the Ia study phase, the early two low doses followed a modified accelerated titration dose escalation design, and the subsequent six doses followed a standard 3 + 3 dose escalation design. MRG003 was administered by intravenous drip on the first day of every three weeks (Q3W). Dose-limiting toxicity (DLT) was assessed on Day 21 (Days 1 to 21) of the first dose administration. The MTD is defined as the dose at which 0/6 or 1/6 subjects in the current dose group experience a DLT, provided that ≥ 33% of subjects experience a DLT at the next higher dose (i.e., 2/3 or 2/6). Even if the MTD has been reached, the selection of the RP2D still requires reference to the MTD and the following available information: low-grade AEs, AEs occurring in subsequent treatment cycles, PK and efficacy data. In the dose escalation phase, if the MTD cannot be determined based on the planned dose (e.g., further dose escalation does not result in a significant increase in exposure), the RP2D will evaluate the available safety, PK, and efficacy data via a dose escalation teleconference or teleconference to determine the dose for the dose expansion phase.<br><br>Once the MTD is determined through a safety assessment meeting, approximately 3 new subjects may be enrolled at the previous low dose level to collect additional PK and safety data upon discussion between the sponsor and the site. At least 6 subjects will be treated at the MTD/RP2D. The MTD/RP2D was selected as the dose for further study in Part Ib.<br><br>In the first-in-human (FIH) study, 6 mg/kg was determined as the highest non-severely toxic dose (HNSTD) in cynomolgus monkeys based on the GLP safety evaluation study of multiple doses of MRG003. According to the

	comprehensive evaluation of the first human dose of ADC drugs by US FDA (Saber-2015), it is considered that the 1/6 of the highest non-severely toxic dose (HNSTD) calculated by body surface area in cynomolgus monkeys is selected as the first in human (FIH) dose, which is an acceptable phase I clinical starting dose under the condition of balanced safety and efficacy. Therefore, it can be speculated that 0.3 mg/kg MRG003 can be used as the starting dose in human clinical trials. However, with reference to the U.S. FDA "Estimating the Maximum Safe Starting Dose for Initial In-Human Exposure" guidelines, the starting dose of MRG003 in human is calculated as 0.2 mg/kg, and for safer consideration, the starting dose of MRG003 is preliminarily set as 0.1 mg/kg. A total of 8 dose levels (0.1, 0.3, 0.6, 1.0, 1.5, 2.0, 2.5, 3.0 mg/kg) were designed. If no MTD is observed at these doses, dose escalation will continue in a 3 + 3 manner by increments of no more than 20% of the previous dose level until the MTD is reached.<br><br>The phase Ia study phase will test the concentration of three drug components in the serum of subjects, i.e., total antibody, conjugated antibody, free small molecule cytotoxin, and analyze the PK parameters of the above three components. In addition, it is also necessary to detect the production of anti-therapeutic drug antibody (ADA) in serum.<br><br>In Phase Ib, a total of 36 eligible subjects with advanced colorectal cancer, head and neck squamous cell carcinoma and nasopharyngeal carcinoma were treated with intravenous drip of MRG003 at MTD/RP2D for 8 cycles in the same dosing regimen as in Phase Ia. To assess the objective response rate (ORR = CR + PR), stable disease (SD), progressive disease (PD), progression-free survival (PFS), overall survival (OS) and duration of response (DOR) of MRG003 on tumors during the treatment and follow-up periods, and to assess the safety, tolerability and kinetics of MRG003.<br><br>Phase I clinical study will provide the final MTD/RP2D dose and PK data and preliminary results of the efficacy and safety of the study drug for phase II clinical trial.<br><br>The Safety Monitoring Committee (SMC) is responsible for safety supervision, dose escalation design, MTD or RP2D, and other important study decisions. However, in case of disagreement between the trial sponsor and the SMC, the decision process will be described in the SMC Charter.
Primary Outcome Measures	Primary Outcome Measures in Part Ia: Subjects will receive different doses of MRG003 by intravenous drip and be observed for dose limiting toxicity (DLT) during the first dosing cycle.<br><br>Part Ib Primary Outcome Measures: To assess the treatment measure related to tumor measurement: objective response rate (ORR) according to RECIST v1.1 criteria.
Secondary outcome measures	Secondary Observation Indicator of Phase Ia:<br><br>• MRG003 was intravenously infused in different dose groups. During corresponding treatment cycles, the concentrations of three drug

	components (total antibody, conjugated antibody, free small molecule cytotoxin) were measured in all subjects, and the PK parameters related to the three drug components were calculated, not limited to Cmax, Tmax, t1/2, MRT, Vd, CL, AUC0-t and AUC0-inf;<br>• To assess the safety and tolerability of the drug, including the type, severity, occurrence time of adverse events, and correlation between adverse events and the study drug;<br>• MRG003 was intravenously infused at different doses to detect drug immunogenicity (ADA);<br>• MRG003 will be administered by intravenous drip at different doses. If the subject does not experience DLT during the dose escalation period (the first dosing cycle), the administration should be continued in the second cycle. Tumor assessments were also performed every 6 weeks.<br>Secondary Observation Indicator of Phase Ib:<br>• Assess the safety and tolerability of the study drug, including the type, severity, occurrence time of adverse events, and correlation between adverse events and the study drug;<br>• At the MTD/RP2D, during the dosing cycle of MRG003, determine the concentrations of three drug components (total antibody, conjugated antibody, free small molecule cytotoxin) in all subjects, and calculate the PK parameters related to the three drug components, not limited to C_{max}, T_{max}, $t_{1/2}$, MRT, V_d, CL, AUC_0-t and $AUC_{0\text{-}inf}$;<br>• Intravenous drip of MRG003 was performed to detect drug immunogenicity (ADA);<br>• Perform tumor assessment according to RECIST v1.1 criteria;<br>• To assess tumor efficacy measures in relation to time: progression-free survival (PFS), overall survival (OS), and duration of response (DOR).
Exploratory Outcome Measures	• To detect the expression level of EGFR in tumor pathological tissues of subjects;
Inclusion criteria	1) Gender: Male or female;<br>2) Age: ≥ 18 years, ≤ 75 years;<br>3) Life expectancy ≥ 12 weeks;<br>4) Phase Ia dose escalation phase: patients with histologically or cytologically confirmed locally advanced or metastatic solid tumors who have failed or cannot obtain standard treatment;<br>5) Phase Ib dose expansion phase: patients with histologically or cytologically

	confirmed locally advanced or metastatic colorectal cancer, head and neck squamous cell carcinoma or nasopharyngeal carcinoma, who have failed standard treatment, cannot tolerate standard treatment or cannot obtain standard treatment;<br><br>6) For patients participating in phase Ib clinical trial, their pathological samples shall be positive for EGFR expression by immunohistochemical detection;<br><br>7) Patients must have at least one measurable lesion as defined by RECIST v1.1;<br><br>8) ECOG score of 0-1;<br><br>9) Patient has organ function meeting the following laboratory test results at baseline examination at the time of enrollment:<br><br>Blood function<br><br>◆ Absolute neutrophil count ≥ 1.5 × 10^9/L;<br><br>◆ Platelets ≥ 100 × 10^9/L;<br><br>◆ Hemoglobin ≥ 9 g/dL;<br><br>Liver and kidney function:<br><br>◆ Total bilirubin ≤ 1.5 × upper limit of normal (ULN);<br><br>◆ ALP ≤ 1.5 times ULN. If the patient has bone metastasis, ALP has no upper limit;<br><br>◆ AST and ALT: ≤ 2.5 times ULN for patients without liver metastasis or ≤ 3 times ULN for patients with liver metastasis;<br><br>◆ International normalized ratio < 1.5 times ULN (anticoagulant naïve) or international normalized ratio < 3 times ULN (anticoagulant naïve);<br><br>◆ Serum creatinine ≤ 1.5 times ULN;<br><br>10) Women of childbearing age must have a negative serum pregnancy test before entering this study and agree to take complete contraception from the beginning of the study to at least 6 months after administration of the study drug; female patients without childbearing potential should have spontaneous amenorrhea for at least 12 months; or have undergone oophorectomy, hysterectomy or tubal ligation more than 6 weeks before screening; in the case of unilateral oophorectomy, their fertility status will be determined by hormone levels;<br><br>11) Male patients must agree to take effective contraceptive measures (male condom with spermicide, or provide evidence of successful vasectomy;

	non-fertile sexual partner, or female partner using intrauterine device with spermicide, female condom with spermicide, contraceptive sponge with spermicide, intravaginal contraception, diaphragm with spermicide, cervical cap with spermicide, or oral/implantable/topical or injectable contraceptives;<br><br>12) Patients voluntarily signed the written informed consent form to participate in this clinical trial, following the study schedule and other requirements of the protocol.
Exclusion Criteria	Patients must not enter the study if any of the following exclusion criteria are fulfilled:<br><br>1) History of allergic reaction to any component of MRG003;<br><br>2) Patients with symptomatic central nervous system (CNS) metastases;<br><br>Note: patients with a previous history of central nervous system metastases who have completed treatment can participate in this trial, that is, patients who have completed treatment for CNS metastases after radiotherapy or surgery 2 weeks before participating in this study and are neurologically stable (no new neurological deficits caused by brain metastases are found in clinical screening and no new lesions are found in central nervous system imaging) can participate in this study. If the patient requires steroids for treatment of CNS metastases, steroids must have been discontinued 2 weeks prior to the first dose;<br><br>3) Patients with a history of other primary malignancies, (except locally resected skin cancer and carcinoma in situ of the cervix, but melanoma needs to be excluded). Patients who previously suffered from other primary tumors but were able to provide treatment for more than 3 years without tumor recurrence or progression can participate in this study;<br><br>4) Patients with a history of clinically significant liver disease, such as hepatitis C (positive hepatitis C antibody test) or chronic active hepatitis B disease (HBsAg positive for more than 6 months, HBV DNA $\geq$ 2000 IU/ml, ALT $\geq$ 2 ULN and excluding hepatitis caused by drugs or other reasons), alcoholic hepatitis, non-alcoholic steatohepatitis, hepatectomy, cirrhosis, etc.;<br><br>5) Patients with known human immunodeficiency virus (HIV) infection;<br><br>6) History of the following ophthalmologic abnormalities, e.g.:<br><br>• Severe dry eye syndrome;<br><br>• Keratoconjunctivitis sicca;<br><br>• Severe exposure keratitis;<br><br>• Any other condition that may result in an increased risk of corneal epithelial damage;

	7) Patient has any serious and/or uncontrolled disease or other condition that, in the opinion of the Investigator and Sponsor, may compromise the patient's participation in this study, as follows:<br>• Uncontrolled diseases, or participation in this study may affect the control of these diseases;<br>• History of interstitial pneumonia, radiation pneumonitis, severe chronic obstructive pulmonary disease, severe pulmonary insufficiency, symptomatic bronchospasm;<br>• Life-threatening autoimmune diseases and ischemic diseases;<br>8) Patients with impaired cardiac function or clinically significant cardiac disease, including any of the following:<br>• Heart rate-corrected baseline QT interval calculated using Fridericia's formula > 450 msec, or congenital QT prolongation syndrome;<br>• Concomitant diseases that may prolong QT interval as assessed by the investigator, such as autonomic neuropathy (due to diabetes or Parkinson's disease), HIV infection, liver cirrhosis, uncontrolled hypothyroidism, or heart failure;<br>• History of severe uncontrolled arrhythmia;<br>• Patients having: myocardial infarction, unstable angina pectoris, coronary artery bypass grafting, congestive heart failure, cerebrovascular accident or transient ischemic attack within 3 months prior to the first dose;<br>9) Use of chemotherapy, biological therapy, immunotherapy, radiotherapy, other clinical studies and other anti-tumor treatment within 4 weeks prior to the first dose; or have not recovered from side effects of the above treatment (CTCAE v4.03 grade 2 or higher). If the half-life of the antineoplastic drug used by the patient is very short (such as fluorouracil), this patient can be appropriately relaxed, which may be allowed to take the drug 2 weeks later. However, this individual case needs to be communicated with and agreed by the sponsor;<br>10) Within 4 weeks before the first administration, major surgery or surgical treatment occurred for any reason.<br>11) The patient has a planned surgery during the study period, or the investigator considers the patient requires surgery;<br>12) History of severe skin diseases requiring interruption of previous EGFR-targeted therapy; or chronic skin diseases requiring oral or intravenous therapy as prescribed;<br>13) The patient has any other concomitant disease that may increase the risk of toxicity as considered by the investigator or sponsor;

	14) Pregnant or lactating.
Route of administration	Intravenous drip
Dose administered	Phase Ia dose escalation phase:<br><br>The starting dose was 0.1 mg/kg, and subsequent dose groups (0.3, 0.6, 1.0, 1.5, 2.0, 2.5, 3.0 mg/kg, with dose escalation not to exceed 20% of the previous dose level until the MTD appeared) followed a standard 3 + 3 dose escalation approach.<br><br>Phase Ib dose expansion phase:<br><br>Maximum tolerated dose (MTD) or recommended Phase II dose (RP2D)
Dosing period	Once every three weeks (Q3W) for up to 8 cycles (24 weeks).
Biomarker Screening Period (Ib applicable)	2 weeks (14 days)
Screening Period	4 weeks (28 days)
Treatment period	Phase Ia: maximum of 8 dosing cycles (24 weeks), each cycle consisting of 21 days;<br><br>Phase Ib: Up to 8 dosing cycles (24 weeks) of 21 days duration.
Post-Treatment Assessments	21 days after the end of the last dosing
Efficacy evaluation follow-up	Visit every 6 weeks until disease progression, start of new antineoplastic therapy, or withdrawal for other reasons or loss to follow-up.
Survival Follow-up	Once every 12 weeks until patient death or loss to follow-up.
MTD or RP2D<br><br>Evaluation	The MTD or RP2D is determined based on the 3 + 3 (modified Fibonacci method) dose escalation criteria and according to the relevant guidelines of the National Cancer Institute, see Section 6.3.4.
PK evaluation	The kinetic parameters of three components of MRG003 (total antibody, conjugated antibody and free small molecule cytotoxin) are not limited to Cmax, Tmax, t1/2, MRT, Vd, CL, AUC0-t and AUC0-inf.
Efficacy evaluation	The tumor response to the study drug treatment will be evaluated according to RECIST 1.1 criteria.
Safety evaluation	• Evaluate adverse events and serious adverse events according to CTCAE v4.03 criteria;

	• To test the immunogenicity of the study drug, anti-therapeutic drug antibodies (ADAs).
Statistical Methods	In this trial, descriptive statistical analysis will be performed on safety indicators, efficacy indicators, PK parameters, immunogenicity indicators and genetic testing, without statistical test.<br>• Safety: The safety and tolerability of MRG003 were assessed by tabulation of adverse events and clinical analysis of laboratory data. The Safety Set (SS) includes all subjects who received at least one treatment.<br>• According to RECIST v1.1 criteria, evaluate the treatment indicators related to tumor measurement: objective response rate (ORR = CR + PR); simultaneously evaluate the tumor response indicators related to time: progression-free survival (PFS), overall survival (OS) and duration of response (DOR); use Kaplan-Meier curve to analyze the overall survival and estimate the survival. Kaplan-Meier curves will also be presented. The log-rank test will be used to compare time-event distributions.<br>• The PK parameters of three components of MRG003 (total antibody, conjugated antibody and free small molecule cytotoxin) were calculated by non-compartmental method for all subjects, but not limited to C_{max}, T_{max}, $t_{1/2}$, MRT, V_d, CL, AUC_{0-t} and AUC_{0-inf}. Number of cases, mean, standard deviation, geometric mean, coefficient of variation, median, minimum, and maximum were calculated for all PK parameters.<br>• Analysis of immunogenicity: including ADA positive rate and ADA.
Ethics	The clinical study protocol (including any changes) and informed consent form must be approved by the Ethics Committee of Sun Yat-sen University Cancer Center in Guangzhou. If this trial involves other clinical study sites, this trial protocol and informed consent form must also be approved by the ethics committee of the respective study site. Written informed consent was obtained from all subjects before entering the trial.
Study period	Estimated screening date of the first patient: 2018.4<br>Estimated completion date of last patient registration: 2020.6
Test period	Approximately 52 months

3 KEY INVESTIGATOR OR STUDY SITE

3.1 Sponsor Responsible

Name, Degree, Title	Chaohong Hu, PhD, CEO
Responsibility	CEO
Name of unit	Shanghai Miracogen Inc.
Address	4E, No. 3, Lane 1238, Zhangjiang Road, Pudong New District, Shanghai
Contact information	021-61637960 ext 8001
Email Address	Mary_hu@miracogen.com.cn

3.2 Principal Investigator

Name, Degree, Title	Ruihua Xu, MD, Professor, President
Responsibility	Clinical Trial Leader
Name of unit	Sun Yat-sen University Cancer Center
Address	651 Dongfeng East Road, Guangzhou
Contact information	020-87343333
Email Address	xurh@sysucc.org.cn

3.3 Safety Monitoring Committee (SMC)

Composition and Head of Safety Monitoring Committee

Clinical unit	Sun Yat-sen University Cancer Center	Ruihua Xu
CRO company	Covance Pharmaceutical R & D (Beijing) Co., Ltd.	Shufang Zhang
Sponsor	Shanghai Miracogen Inc.	Medical Department

3.4 Central Laboratory

Name of unit	Guangzhou KingMed Center for Clinical Laboratory
Address	No. 10, Luoxuan 3rd Road, Guangzhou International Biological Island
Name of unit	Covance Bioanalytical Laboratory
Address	Building 3, No. 3377, Zhoupu Kangxin Road, Pudong, Shanghai

4 INTRODUCTION: STUDY BACKGROUND AND BASIS

4.1 Background information

4.1.1 Solid tumor background

EGFR is a member of the human epidermal growth factor receptor (HER) family, which is widely distributed on the surface of mammalian epithelial cells, fibroblasts, glial cells, keratinocytes and other cells. Its signaling pathway plays an important role in physiological processes such as cell growth, proliferation and differentiation. Overexpression or mutation of EGFR will cause tumors. Studies have shown that EGFR is overexpressed or abnormally expressed in a variety of malignant cells of epithelial origin, such as colorectal cancer, esophageal cancer, head and neck squamous cell carcinoma, gastric cancer, breast cancer, prostate cancer, bladder cancer, renal cancer, pancreatic cancer and ovarian cancer (Arteaga-2002). EGFR is expressed in a variety of human tumors, with approximately 25% -77% of colorectal cancers, 80% -100% of head and neck cancers, 40% -80% of non-small cell lung cancers, and 30% -50% of pancreatic cancers having EGFR expression (Herbst-2002). At present, the preliminary proposed indications of MRG003 are solid tumors with positive EGFR expression, including colorectal cancer, head and neck squamous cell carcinoma and nasopharyngeal carcinoma.

4.1.2 Background of relevant therapeutic drug

Based on the correlation between EGFR expression and the development of cancer, scientists have developed multiple EGFR target inhibitors in recent years and clinically validated their anti-tumor activity. Currently marketed EGFR target inhibitors mainly include small molecule tyrosine kinase inhibitors (TKIs) and monoclonal antibodies (mAbs), as shown in. EGFR single-target inhibitors mostly target lung cancer, colorectal cancer, breast cancer and pancreatic cancer with high EGFR expression; antibody drugs mainly treat colorectal cancer, head and neck cancer and nasopharyngeal cancer with high EGFR expression.

Table: Marketed EGFR Target Drugs

Type	Name	Target/Type	Indications for Use	Approval date	Company
Small molecule tyrosine kinase inhibitor	Gefitinib	EGFR	NSCLC	2003	AstraZeneca
	Erlotinib	EGFR	NSCLC, pancreatic cancer	2004/2005	OSI, Roche, Genentech
	Lapatinib	EGFR, ErB2	Breast cancer	2007	GSK
	Icotinib	EGFR	NSCLC	2011	Betta
	Afatinib	EGFR, ErB2/4	NSCLC	2013	Boehringer Ingelheim
Monoclonal	Cetuximab	EGFR/Chimera	Colorectal cancer, head and neck	2004/2006	Merck KGaA

antibody			cancer		
	Panitumumab	EGFR/humanized	Colorectal Cancer	2006	Amgen
	Nimotuzumab	EGFR/humanized	Nasopharyngeal carcinoma	2008	Baitai Biological Pharmaceutical

4.2 Basis for Study

4.2.1 Limitations of available treatments

At present, the treatment of solid tumors is mainly surgical resection and medical treatment. Drug therapy mainly includes traditional chemotherapeutic drugs and targeted drugs. Targeted drug therapy is to design corresponding therapeutic drugs at the cellular and molecular levels, based on the mechanism of tumorigenesis, for specific treatment of tumors. At present, scientific research has found a number of targets related to tumorigenesis, of which EGFR is a receptor tyrosine kinase target. The mutation or overexpression of EGFR is related to the occurrence and development of tumors. Studies have shown that there is high or abnormal expression of EGFR in a variety of malignant solid tumor cells of epidermal origin. Therefore, EGFR has become a hot target for anti-tumor drug research and development.

At present, the clinical therapeutic drugs targeting EGFR mainly include small molecule tyrosine kinase inhibitors (TKIs) (gefitinib, erlotinib, etc.) targeting the receptor cells, and monoclonal antibodies (e.g., Cetuximab, panitumumab) targeting the EGFR receptor (Di-2013). Traditional chemotherapy has a definite effect on some tumors, but its toxic and side effects reduce the patient's tolerance.

Gefitinib, Icotinib, and Erlotinib are small molecule tyrosine kinase inhibitors that have been approved for the treatment of metastatic lung cancer after failure of chemotherapy. Compared with basic combination chemotherapy regimens, such as: Docetaxel + Cisplatin (DP), Gemcitabine + Cisplatin (GP), Paclitaxel + Cisplatin (TP), Vinorelbine + Cisplatin (NP), their efficacy is equivalent, safety is higher than chemotherapy drugs, but they also have more toxic side effects, such as rash, diarrhea and so on. The main problem of this class of small molecule tyrosine kinase inhibitors is that they are prone to primary or secondary drug resistance. Data show that about 50% of patients with cell lung cancer will develop acquired drug resistance after treatment with gefitinib and erlotinib, so their clinical application is limited (Yang Yaqiong-2012). Although there is a theoretical possibility of targeted EGFR therapy, the efficacy of anti-EGFR therapy is not significant (Yangyagon-2012). Afatinib is an irreversible EGFR-HER2 dual tyrosine kinase receptor inhibitor that overcomes EGFR-TKI resistance to gefitinib and erlotinib. In terms of efficacy, compared with the placebo group, the survival rate of patients in Afa group showed no advantage, but it could significantly prolong the progression-free survival (OS, 10.8 months: 12.0 months; PFS, 3.3 months: 1.1 months) of patients; compared with chemotherapy, it could also significantly improve the progression-free survival of patients. It is a promising small molecule kinase inhibition for the

treatment of NSCLC, with the main adverse reactions similar to other small molecule inhibitors with the same target. There are other indications under clinical study (Wang Caixia 2015).

Cetuximab, as a monoclonal antibody drug, is a human-mouse chimeric IgG1 anti-EGFR monoclonal antibody. In 2004, the US FDA approved Cetuximab for the treatment of colorectal cancer. In 2006, Cetuximab was approved for the treatment of head and neck cancer. Currently, there are more clinical trials for other tumor indications. Compared with chemotherapeutic drugs, antibody drugs have the characteristics of strong specificity and less side effects, and have achieved good clinical efficacy. In the treatment of colorectal cancer, Cetuximab can increase the therapeutic effect of irinotecan (CPT-11) -based chemotherapy, its combination with chemotherapy has a higher efficacy than treatment, and Cetuximab can overcome the drug resistance of CPT-11, but there is no significant difference in overall survival between Cetuximab alone and in combination with irinotecan in patients with refractory colorectal cancer with EGFR expression who have failed fluorouracil and CPT-11 treatment [overall survival (OS) 6.9 months, 8.6 months, respectively], and the overall response rate (ORR) is low [overall response rate (ORR) 10.8%, 16.4%, respectively]. With the optimization of medication regimen, recently, in the first-line treatment of unresectable metastatic colorectal cancer, the combination of Cetuximab and FOLFIRINOX (fluorouracil + leucovorin + irinotecan + oxaliplatin) achieved the highest response rate reported so far (80.9%), with survival and progression-free survival of 24.7 months and 9.5 months, respectively (Blick-2007, Yangyagon-2012). These results indicate that Cetuximab should be used in combination with chemotherapeutic drugs to improve the efficacy, and its efficacy alone.

Panitumumab is a fully humanized IgG2 anti-EGFR monoclonal antibody prepared using transgenic mouse technology and approved by the FDA in September 2006 for marketing in combination with fluoropyrimidine, oxaliplatin, and irinotecan or after chemotherapy for the treatment of EGFR-positive metastatic colorectal cancer. In 2006, the FDA approved its treatment for chemotherapy-resistant metastatic colorectal cancer. Although the antitumor activity and safety of panitumumab have been clinically demonstrated, there is no significant difference in the overall survival time of patients between panitumumab and best supportive care (BSC) (Xu Ruihua-2010). Panitumumab has no significant advantage over the chimeric antibody Cetuximab in clinical efficacy. The efficacy is not significant when used alone, and it needs to be used in combination with chemotherapeutic drugs in clinical treatment.

At present, the problems of EGFR targeted therapy drugs include: 1) Patients develop drug resistance to therapeutic drugs during chemical drug therapy. Data show that 50% of patients with advanced cell lung cancer develop drug resistance (Kosaka-2011). 2) KRAS gene mutation leads to no therapeutic effect of antibody drugs. KRAS gene mutations have been reported in 40% to 50% of colorectal cancer patients (Kahlenberg-2003). Based on this estimate, there are approximately 612,000 colorectal cancer patients worldwide and 112,000 colorectal cancer patients in China each year without effective therapeutic agents.

From this point of view, the current treatment methods and drugs are still far from meeting the needs of clinical treatment, and there is a great unmet clinical need. Therefore, it is necessary to develop more targeted and effective targeted therapeutic drugs to achieve individualized treatment of cancer patients.

As an ADC drug, MRG003 has the potential to overcome the ineffectiveness of antibody-targeted therapy caused by EGFR mutants and KRAS mutants due to its mechanism of action different from that of monoclonal antibodies. This inference has been preliminarily confirmed in preclinical in vitro and in vivo pharmacodynamic studies with MRG003.

4.2.2 Study drug

4.2.2.1 <u>Drug name: MRG003 for Injection</u>

Drug structure: MRG003 is composed of three parts: 1) anti-EGFR monoclonal antibody; 2) potent cytotoxic small molecule, monomethyl auristatin E (MMAE); 3) protease degradable linker monoclonal antibody and small molecule drug, valine-citruline (vc) Linker. MRG003 is produced by the conjugation reaction of chemically synthesized linker-drug vcMMAE with monoclonal antibody.

Principle of action of the drug: MRG003 is a conjugate formed by IgG1 monoclonal antibody against EGFR and vcMMAE. Based on the mechanism of action of ADC, shown in Table 1, MRG003 specifically recognizes and binds EGFR on the surface of tumor cells, enters the cell through EGFR-mediated endocytosis, and releases MMAE through lysosomal endoproteinase degradation in the cell; after entering the cytoplasm, MMAE binds tubulin and inhibits tubulin polymerization, thereby blocking various cell physiological functions involved in tubulin, including mitosis, thereby inhibiting tumor cell proliferation and leading to tumor cell death.

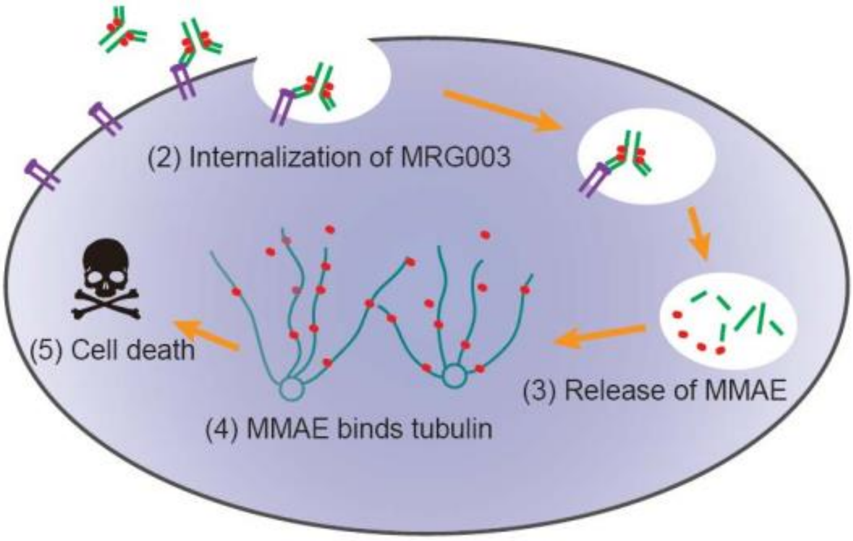

Figure 1: Principle of therapeutic effect of antibody-conjugated drugs

4.2.2.2 <u>Major metabolic pathways</u>

The absorption, metabolism, distribution and excretion (ADME) characteristics of antibody-conjugated drugs (ADC) in organisms mainly include the stability of linker in plasma, the tissue distribution and tissue accumulation of ADC, metabolic pathways, the biological activity of metabolites and metabolites.

ADC drugs are composed of targeting antibodies and cytotoxic small molecules connected by a linker. In view of the molecular structure characteristics of ADC, its biodistribution is similar to that of IgG antibody. The antibody decomposition and elimination process in ADC is similar to that of monoclonal antibody. According to the literature reports (Lin-2013, Han-2014), the metabolism and elimination of antibody mainly occur in the liver, spleen, lymph nodes, intestine and kidney, and finally degrade into amino acids or peptides without biological activity. ADC is mainly transported into lysosomes by receptor-mediated endocytosis or pinocytosis and then degraded by enzymes, so the swallowed ADC antibody part has been basically degraded into amino acids or biologically inactive peptides in lysosomes. The typical metabolic process of ADC is shown in 2. (Fig. Lin-2013)

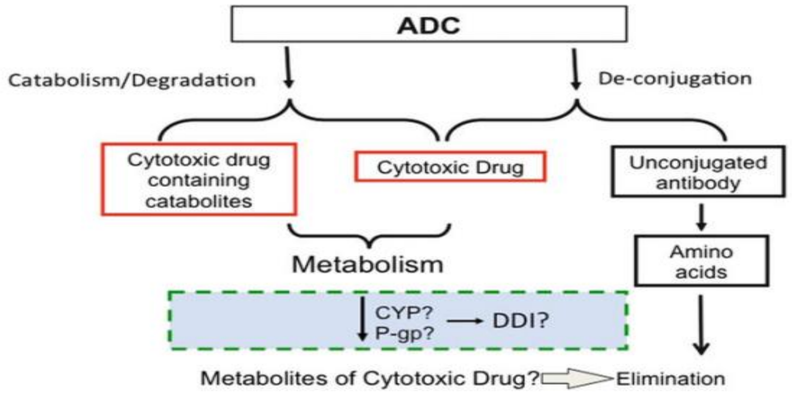

Fig. 4 Diagram of theoretical ADC catabolism. The formation of cytotoxic drug-containing products from ADCs may occur by two concurrent processes: deconjugation and catabolism. The deconjugation process includes release of cytotoxic drug-containing products from the ADC via enzymatic or chemical processes and unconjugated antibody, with preservation of the antibody backbone. The catabolism process includes proteolytic catabolism of the antibody and formation of cytotoxic drug-containing catabolites (free drug or drug–amino acid conjugates)

Figure 2: Typical Metabolic Process of ADC

MRG003 is administered as an intravenous infusion, after which peak plasma concentrations are rapidly achieved. PK/PD studies of xenografts showed that MRG003 was quickly absorbed by the targeted tissue and subsequently enriched in the targeted tissue after entering the blood, and MMAE was enzymatically released.

According to the literature (Lin-2013), the biodistribution of ADC is similar to the distribution of IgG antibody. Since both MRG003 and Cetuximab are IgG1 antibodies against EGFR with the same target, it can be preliminarily inferred that MRG003 has similar tissue distribution to Cetuximab.

Experimental results on the cross-reactivity of Cetuximab with mouse, rat, cynomolgus monkey and human tissues showed that Cetuximab only bound to the epithelial cells of cornea, placenta, prostate, liver, lung bronchus, esophagus, mammary gland and salivary gland of human and monkey eyes, as well as the linear and squamous epithelial cells of skin, but did not bind to the tissues of mouse and rat (FDA Erbitux ® Pharmacology Review). This was generally consistent with the results of tissue cross-reactivity test of MRG003 in normal subjects and cynomolgus monkeys.

Metabolism and Excretion of MMAE In the marketed Adcetris ® studies, Adcetris ® was shown to be highly stable, with only a very small fraction of extracellular MMAE released from the ADC and metabolized, producing free drug as well as concentrations insufficient to cause cytotoxicity.

The results of in vitro [^{3}H] -MMAE metabolic phenotyping study showed that [^{3}H] -MMAE was converted into eight radioactive substances by human liver microsomal NADPH. Metabolites are produced primarily by O-demethylation, N-demethylation, and dehydrogenation and are mediated by CYP3A4, with other CYP450 enzymes likely involved in the formation of only a small fraction.

In the study of in vitro determination of the metabolic characteristics of [^{3}H] -MMAE in liver microsomes from different animal species and humans, rat, monkey, and human hepatocytes were incubated with 10 μM [^{3}H] -MMAE for 0, 60, 120, and 240 min, respectively, and a total of 12 compounds were found, and the migration of molecular weight was mainly through hydroxylation, demethylation, dehydrogenation, or hydrolysis. Among them, all 12 compounds were detected in monkey hepatocytes, and 9 compounds with slight differences were detected in rats and humans.

Inhibition of human hepatic cytochrome P450 enzymes by MMAE experiments primarily determined the inhibitory effect of MMAE on major CYP enzymes (CYPIa2, CYP2B6, CYP2C8, CYP2C9, aCYP2C19, CYP2D6, CYP3A4/5). In the experiment, human liver microsomes were incubated with different concentrations of MMAE (0.1-100 μM). The results showed that MMAE could directly inhibit CYP3A4/5 through midazolam 1 'hydroxylation at IC50 of 10 μM, and testosterone 6β-hydroxylation had little or no direct inhibition on CYPIa2, CYP2B6, CYP2C8, CYP2C9, CYP2C19, CYP2D6 or CYP3A4/5 at IC50 > 100 μM. MMAE inhibited CYP3A4/5 in a time-dependent manner.

The results of the excretion test of Adcetris® in rats showed that after intravenous administration of 3 mg/kg Adcetris ® (cAC10-vc-3H-MMAE) or 0.056 mg/kg 3H-MMAE, urine and feces were collected at intervals of 24 h (up to 672 h) from 0 to 12 h and 12 to 24 h. The concentrations of radioactivity in blood, plasma, urine, feces, and carcasses were determined by LS, the types of emitted substances in urine and feces were determined by HPLC/UV, and the main metabolites were detected by LC-MS/MS. The results showed that the main elimination pathway of cAC10-vc-3H-MMAE and 3H-MMAE in rats was through feces, and part of it was excreted through urine. Approximately 89% (males) or 97% (females) of the cAC10-vc-3H-MMAE radioactivity was detected in feces and

approximately 14% (males) or 7% (females) in urine. Of the 3H-MMAE, approximately 97% (males) or 102% (females) of the radioactivity was detected in feces, and approximately 15% (males) or 9% (females) of the radioactivity was detected in urine. In summary, the metabolic pattern and metabolites of MMAE were similar in rat, monkey, and human liver microsomes, and MMAE inhibited CYP3A4/5 but not other CYP isoforms. MMAE did not induce CYP450 enzymes in human hepatocytes. Metabolism of MMAE occurs primarily through oxidation by CYP3A4/5. MMAE is excreted primarily in the feces and partially in the urine, primarily as unchanged MMAE (Adcetris ® FDA Pharmacology Review).

In addition, in a clinical human MMAE metabolism study, it was found that approximately 72% of MMAE recovered in feces after Adcetris® was intravenously infused at 1.8 mg/kg for one week, which was consistent with the results of preclinical studies. (Adcetris® Labeling)

5 TRIAL OBJECTIVE

5.1 Primary Trial Objectives

The primary objectives of the Phase Ia dose-escalation study are:

- Determine the maximum tolerated dose (MTD) or recommended Phase II dose (RP2D) of MRG003.

The primary objectives of the Phase Ib dose expansion phase of the study are:

- To assess the antitumor activity of MRG003 in patients with advanced colorectal cancer, head and neck squamous cell carcinoma, and nasopharyngeal carcinoma.

5.2 Secondary Trial Objectives

Secondary objectives of the Phase Ia dose-escalation study:

- To evaluate the kinetic characteristics of the drug in patients with advanced solid tumors by kinetic study;
- To observe the safety of MRG003 in patients with advanced solid tumors;
- To assess the immunogenicity of MRG003 in patients with advanced solid tumors;
- To assess the antitumor activity of MRG003 in patients with solid tumors.

Secondary objectives of the Phase Ib dose expansion phase of the study:

- To evaluate the safety and tolerability of MRG003 in patients with advanced colorectal cancer, head and neck squamous cell carcinoma and nasopharyngeal carcinoma;
- Evaluate the kinetic characteristics of MRG003;
- To evaluate the immunogenicity of MRG003 in patients with advanced colorectal cancer, head and neck squamous cell carcinoma and nasopharyngeal carcinoma.

5.3 Explore trial objectives

- To explore the correlation between EGFR expression in tumor pathological tissues of subjects and efficacy indicators.

6 TRIAL DESIGN AND OBSERVATION INDICATORS

6.1 Total trial design

This study is designed as two parts: the first part of Phase Ia dose escalation study is a single-center, non-randomized, single-arm, open-label clinical trial for the first time in human to determine the maximum tolerated dose (MTD) or recommended Phase II dose (RP2D) of MRG003 in patients with advanced solid tumors; the second part of Phase Ib dose expansion study is a multi-center, non-randomized, single-arm, open-label clinical trial to preliminarily evaluate the efficacy of MRG003 in patients with advanced colorectal cancer, head and neck squamous cell carcinoma and nasopharyngeal carcinoma. Both parts will evaluate the safety, tolerability, immunogenicity and kinetics of MRG003 in human.

In the Ia study phase, the early two low doses followed a modified accelerated titration dose escalation design, and the subsequent six doses followed a standard 3 + 3 dose escalation design. MRG003 was administered by intravenous drip on the first day of every three weeks (Q3W). Dose-limiting toxicity (DLT) was assessed on Day 21 (Days 1 to 21) of the first dose administration. The MTD is defined as the dose at which 0/6 or 1/6 subjects in the current dose group experience a DLT, provided that ≥ 33% of subjects experience a DLT at the next higher dose (i.e., 2/3 or 2/6). Even if the MTD has been reached, the selection of the RP2D still requires reference to the MTD and the following available information: low-grade AEs, AEs occurring in subsequent treatment cycles, PK and efficacy data. In the dose escalation phase, if the MTD cannot be determined based on the planned dose (e.g., further dose escalation does not result in a significant increase in exposure), the RP2D will evaluate the available safety, PK, and efficacy data via a dose escalation teleconference or teleconference to determine the dose for the dose expansion phase. Once the MTD is determined through a safety assessment meeting, approximately 3 new subjects may be enrolled at the previous low dose level to collect additional PK and safety data upon discussion between the sponsor and the site. At least 6 subjects will be treated at the MTD/RP2D. The MTD/RP2D was selected as the dose for further study in Part Ib.

In the first-in-human (FIH) study at MTD/RP2D, according to the comprehensive evaluation on the first-in-human (Saber-2015) dose of ADC by US FDA, it is considered that one-sixth of the highest non-severely toxic dose (HNSTD) calculated by body surface area in cynomolgus monkeys is selected as the first-in-human (FIH) dose, which is an acceptable initial dose for phase I clinical study under the condition of balanced safety and efficacy. Therefore, it can be speculated that 0.3 mg/kg MRG003 can be used as the initial dose in human clinical trials. However, with reference to the U.S. FDA Guideline on Estimating the Maximum Safe Starting Dose for Initial In-Human Clinical Trials, the calculated starting dose of MRG003 in human clinical trials is 0.2 mg/kg, and for safer consideration, the starting dose of MRG003 is preliminarily set as 0.1 mg/kg. A total of 8 dose levels (0.1, 0.3, 0.6, 1.0, 1.5, 2.0, 2.5, 3.0 mg/kg) were designed. If no MTD is

observed in these dose groups, dose escalation will continue in a 3 + 3 manner by escalating no more than 20% of the previous dose level until the MTD is reached.

In the phase Ia study, the concentrations of three components of MRG003 in the systemic circulation of subjects, total antibody, conjugated antibody and unconjugated drug, will be detected, and the PK parameters of the above three components will be analyzed. In addition, it is also necessary to detect anti-drug antibody.

In Phase Ib, a total of 36 eligible subjects with advanced colorectal cancer, head and neck squamous cell carcinoma and nasopharyngeal carcinoma were treated with intravenous drip of MRG003 at MTD/RP2D for 8 cycles in the same dosing regimen as in Phase Ia. To assess the objective response rate (ORR = CR + PR), stable disease (SD), progressive disease (PD), progression-free survival (PFS), overall survival (OS) and duration of response (DOR) of MRG003 on tumors during the treatment and follow-up periods, and to assess the safety, tolerability and kinetics of MRG003.

Phase I clinical study will provide the final MTD/RP2D dose and PK data and preliminary results of the efficacy and safety of the study drug for phase II clinical trial.

The Safety Monitoring Committee (SMC) is responsible for safety supervision, dose escalation design, MTD or RP2D, and other important study decisions. However, in case of disagreement between the trial sponsor and the SMC, the decision process will be described in the SMC Charter.

6.2 Observation indicators

6.2.1 Primary Outcome Measures

Primary Outcome Measures in Part Ia: Subjects will receive different doses of MRG003 by intravenous drip and be observed for dose limiting toxicity (DLT) during the first dosing cycle.

Primary Outcome Measures for Part Ib: Assessment of treatment measures related to tumor measurement according to RECIST v1.1 criteria: Objective response rate (ORR)

6.2.2 Secondary outcome measures

Secondary Outcome Measures in Part Ia:

- MRG003 was intravenously infused in different dose groups. During the treatment period, the concentrations of the three drug components were determined in all subjects, and the PK values of the three drug components were calculated, not limited to C_{max}, T_{max}, $t_{1/2}$, MRT, V_d, CL, $AUC_{0\text{-}t}$ and $AUC_{0\text{-}inf}$;
- To assess the safety and tolerability of the drug, including the type, severity, occurrence time of adverse events, and correlation between adverse events and the study drug;

- MRG003 was intravenously infused at different doses to detect drug immunogenicity (anti-therapeutic drug antibody, ADA);
- MRG003 will be administered by intravenous drip at different doses. If the subject does not experience DLT during the dose escalation period (the first dosing cycle), the administration should be continued in the second cycle. Tumor assessments were also performed every 6 weeks.

Secondary Outcome Measures in Part Ib:

- Assess the safety and tolerability of the study drug, including the type, severity, occurrence time of adverse events, and correlation between adverse events and the study drug;
- At MTD/RP2D, during the treatment cycle of intravenous drip of MRG003, determine the concentrations of the three drug components in all subjects, and calculate the PK values of the three drug components, not limited to C_{max}, T_{max}, $t_{1/2}$, MRT, V_d, CL, AUC_{0-t} and AUC_{0-inf};
- Intravenous drip of MRG003 to detect drug immunogenicity (ADA)
- Perform tumor assessment according to RECIST v1.1 criteria;
- To assess time-related tumor response measures: progression-free survival (PFS), overall survival (OS) and time to tumor response (DOR);

6.2.3 Exploratory Outcome Measures

- To detect the expression level of EGFR in tumor pathological tissues of subjects;

6.3 Drug tolerability assessment

6.3.1 Selection of starting dose

In the first-in-human Phase I clinical trial, the preliminary starting dose of MRG003 was set at 0.1 mg/kg administered once every 3 weeks (Q3W). According to the multiple dose GLP safety evaluation study of MRG003 in cynomolgus monkeys, the doses of 1, 3 and 6 mg/kg were administered once every three weeks for 4 doses (Q3W4), and the study results determined that 6 mg/kg was the HNSTD in cynomolgus monkeys. According to the comprehensive evaluation of the first human dose of ADC drugs by US FDA (Saber-2015), it is considered that the dose 1/6 of the highest non-severely toxic dose (HNSTD) calculated by body surface area in cynomolgus monkeys is selected as the first in human (FIH) dose, which is an acceptable phase I clinical starting dose under the condition of balanced safety and efficacy. Therefore, it is speculated that 0.3 mg/kg MRG003 can be used as the starting dose in human clinical trials. However, with reference to the US FDA Guidelines for Estimating the Maximum Safe Starting Dose for the First Time in Human,

the calculated starting dose of MRG003 in human is 0.2 mg/kg. For safer consideration, the initial starting dose of MRG003 can be set at 0.1 mg/kg.

6.3.2 Selection of maximum dose

The maximum dose that may be achieved during the dose escalation phase is 3.0 mg/kg. According to several clinical trials of vcMMAE-based ADC drugs for different targets in Europe and the United States, DLT is usually caused by the toxicity of MMAE, and MTD is generally occurred at the dose of 1.8 – 2.4 mg/kg.

6.3.3 Dose Escalation Scheme

MRG003 will be assessed for dose-limiting toxicity during the first treatment cycle of Phase Ia. Intravenous drip of a certain dose of MRG003 was given to the patient for recuperation for 3 weeks, which was considered as 1 treatment cycle. In the drug tolerance study, the modified accelerated titration dose escalation method was followed for the early two dose groups (0.1, 0.3 mg/kg), and the standard 3 + 3 (modified Fibonacci method) dose escalation method was followed for the subsequent dose groups (0.6, 1.0, 1.5, 2.0, 2.5, 3.0 mg/kg). The escalated doses and the number of subjects are presented in Table 2. In the 0.1 mg/kg and 0.3 mg/kg dose periods, only 1 subject was required. If a subject experiences an adverse reaction at 0.1 mg/kg and meets the DLT criteria, the tolerance test protocol will be converted to the 3 + 3 method (i.e., add another 2 subjects in the 0.1 mg/kg dose group); if the adverse reaction of a subject does not meet the DLT criteria, the tolerance test can enter the next dose group. Doses of 0.6 mg/kg and higher were escalated by the 3 + 3 method. In any dose group, if none of the 3 subjects experienced DLT at the end of treatment cycle, the tolerance test could enter the next higher dose group; if 1 of the 3 subjects experienced DLT at the end of treatment cycle, another 3 subjects were included in this dose group. If none of the additional 3 subjects experienced DLT, the tolerance test could enter the next dose group. If 2 or more of 3 or 6 subjects in a dose group experienced DLT, dose escalation was stopped and the previous low dose group was the maximum tolerated dose (MTD). To find a more accurate MTD, an intermediate dose group may be added to the preset dose based on available safety and PK data. Dose escalation plan see Table 2. If the MTD is not observed in the above dose groups, dose escalation will continue in a 3 + 3 manner by escalating no more than 20% of the previous dose level until the MTD is reached. In the 3 + 3 escalation method, each subject received only one corresponding dose. The next escalating dose was not entered until the end of each dose observation. Trials with more than two dose groups could not be performed simultaneously. See Table 3 for the judgment principles of MTD or RP2D.

Table 2: Dose escalation scheme and number of subjects

Dose Group	**1**	**2**	**3**	**4**	**5**	**6**	**7**	**8**	**9 above**
Escalation		200%	100%	67%	50%	33%	25%	20%	< 20%
Dose (mg/kg)	0.1	0.3	0.6	1.0	1.5	2.0	2.5	3.0	TBD
Number of people	1	1	3 + 3	3 + 3	3 + 3	3 + 3	3 + 3	3 + 3	3 + 3

Tolerance	√	√	√	√	√	√	√	√	√
Kinetics	√	√	√	√	√	√	√	√	√

Table 3: 3 + 3 dose escalation method and MTD and RP2D determination principles

Number of subjects meeting DLT criteria at a dose	Dose escalation principle
No subjects met the criteria for DLT	Three new subjects entered the next dose phase
3 subjects 2 or 3 met criteria for DLT	The tolerability trial was terminated. The dose of the former group was the MTD. If only 3 subjects are tested in the previous dose group, another 3 new subjects shall be selected to enter the previous dose group for the study to explore MTD/RP2D dose.
3 subjects – 1 met criteria for DLT	An additional 3 new subjects should be re-tested at this dose stage.<br><br>If no newly selected 3 patients meet the criteria for DLT, another 3 patients can be selected to enter the dose phase for the trial.<br><br>If 1 or more of the newly selected 3 patients meet the criteria for DLT, the tolerance test is terminated, and the dose of the previous group is the MTD. If only 3 subjects are tested in the previous dose group, another 3 new subjects shall be selected to be tested in the previous dose group (0/6 or 1/6).
The RP2D was selected with reference to the MTD and the following information available: low-grade AEs, AEs occurring in subsequent treatment cycles, PK, and efficacy data.	At least 6 subjects were treated at the MTD/RP2D. The MTD/RP2D dose will be assessed by the Safety Monitoring Committee (SMC).

6.3.4 Dose-limiting toxicity, maximum tolerated dose and recommended phase II dose

6.3.4.1 Judgment criteria for dose limiting toxicity (DLT)

A DLT was defined as an AE (except allergic reaction) or abnormal laboratory value not related to disease progression, or concomitant disease, or concomitant medication, and met any of the following criteria in the DLT table (Table 4). Adverse events will be assessed according to CTCAE v4.03. Any subject who fails to complete the 21-day observation period for reasons other than DLT will be considered as not evaluable for DLT, and another subject in the same dose group should be added.

Table 4: Judgment criteria for DLT according to CTCAE 4.03 classification

Toxicity	Any of the following criteria
Blood	Grade 4 neutropenia lasting > 7 consecutive days
	Grade 3 thrombocytopenia lasting > 7 consecutive days
	Grade 4 thrombocytopenia
	Grade 3 febrile neutropenia (granulocytes < 1.0 × 109/L, body temperature ≥ 38.5°C) requiring antibiotics
Kidney	Serum creatinine greater than 3 times the upper limit of normal for > 7 consecutive days
	≥ Grade 3: Blood creatinine increased
Liver	Total bilirubin greater than 3 times the upper limit of normal for > 7 consecutive days
	≥ Grade 3 total bilirubin elevation
	Grade 3 AST or ALT elevation lasting > 7 consecutive days
	Grade 4 elevated aspartate aminotransferase (AST) or alanine aminotransferase (ALT)
Pancreas	Symptomatic amylase or lipase abnormalities ≥ CTCAE Grade 3
	Asymptomatic ≥ CTCAE Grade 3 amylase or lipase abnormality lasting longer than 7 days
Cardiovascular system	≥ Grade 3
Eye	≥ Grade 3
Skin	Grade 3 or 4 skin toxicities that remain uncontrolled despite symptomatic treatment
Nervous system	≥ Grade 3: Neurologic toxicity
Other Adverse Events	≥ Grade 3: Uncontrolled vomiting and nausea despite the use of antiemetic doses
	≥ Grade 3: Uncontrolled diarrhea despite the subject's use of antidiarrheal medication
	Treatment delay of > 14 days due to unresolved toxicity that does not resolve to Grade 1 or baseline
	Other ≥ Grade 3 AE

	Any other unacceptable toxicity as determined jointly by the investigator and the sponsor's medical monitor
Note: 3 kinds of adverse reactions are not regarded as criteria for DLT judgment: ≥ Grade 3 anemia (unless there is indirect relationship with MRG003); ≥ Grade 3 lymphocytopenia (unless there is clinical significance); ≥ Grade 3 hypercholesterolemia or hypertriglyceridemia.	

6.3.4.2 Determine MTD or RP2D

The MTD is defined as the dose at which 0/6 or 1/6 subjects in the current dose group experience a DLT, provided that ≥ 33% of subjects experience a DLT at the next higher dose (i.e., 2/3 or 2/6). Even if the MTD has been reached, the selection of the RP2D still requires reference to the MTD and the following available information: low-grade AEs, AEs occurring in subsequent treatment cycles, PK and efficacy data. In the dose escalation phase, if the MTD cannot be determined based on the planned dose (e.g., further dose escalation does not result in a significant increase in exposure), the RP2D will evaluate the available safety, PK, and efficacy data via a dose escalation teleconference or teleconference to determine the dose for the dose expansion phase.

Once the MTD has been determined through a safety assessment meeting, additional approximately 3 subjects may be enrolled at the previous low dose level to collect additional PK and safety data upon discussion between the sponsor and the site. At least 6 subjects will be treated at the MTD/RP2D. The MTD/RP2D was selected as the dose for further study in Part Ib.

6.3.5 SMC Responsibilities

The Safety Monitoring Committee (SMC) will be responsible for safety supervision, dose escalation design, determination of MTD or RP2D, and other important study decisions. However, in case of disagreement between the trial sponsor and the SMC, the decision process will be described in the SMC Charter.

6.4 Evaluation of Kinetics

In the clinical study, the concentrations of three drug components of MRG003 in the systemic circulation of subjects, total antibody, conjugated antibody and unconjugated drug, as well as anti-drug antibody, will be detected. Before, during and after intravenous drip of MRG003, blood samples will be collected from all subjects at set time points, and PK parameters (not limited to C_{max}, T_{max}, $t_{1/2}$, MRT, V_d, CL, $AUC_{0\text{-}t}$ and $AUC_{0\text{-}inf}$, etc.) of the above components in serum will be analyzed.

Refer to Table 7 for PK and ADA serum sample collection points. Blood samples will be collected from vein during blood collection. The test items, blood volume and treatment are shown in Table 8. The blood collection time should be recorded in detail in the eCRF

to minute. If other blood collection time points coincide with PK blood collection time points, PK blood collection should be performed first.

6.5 Efficacy Assessments

Response to tumor therapy will be assessed in this study using Response Evaluation Criteria in Solid Tumors RECIST v1.1, see Appendix II.

The primary outcome measure of Ib trial is objective response rate (ORR), the proportion of subjects with confirmed overall CR and confirmed overall PR in all subjects who have received the study drug.

Progression-free survival (PFS) for this study is defined as the time from the start of treatment with MRG003 until objective tumor progression or death from any cause. Duration of response (DOR) is defined as the time from the first documented response until disease progression or death among subjects with a confirmed objective response (CR or PR), with tumor evaluation according to RECIST v1.1 criteria. Overall survival (OS) is defined as the time from the start of treatment with MRG003 to death due to any cause.

For patients without disease progression after the last dosing cycle, tumor response should be assessed every 6 weeks and followed up for survival until disease progression or other anti-tumor treatment. Survival follow-up could be performed every 12 weeks after confirmation of disease progression or receipt of additional antineoplastic therapy. The follow-up period starts from the end of treatment cycle until death or study termination. During follow-up, survival status and subsequent tumor treatment need to be examined and recorded. Subsequent tumor treatment does not need to be recorded in electronic case report form (eCRF).

Tumor evaluation will be performed according to RECIST v1.1, and the results of imaging assessment methods should be recorded in the electronic case report form. During trial treatment, radiographic assessments may be performed ± 7 days from the scheduled date and the methodology should be consistent between baseline and subsequent follow-up. Whenever clinically permissible, the same oral or intravenous contrast agent was used for each scan, and, whenever possible, the same investigator/radiologist evaluated all patient scans during the study.

7 TRIAL, WITHDRAWAL, DISCONTINUATION

7.1 Number of subjects

Part Ia: Approximately 26 patients with solid tumors will participate in the dose escalation study to determine MTD/RP2D;

Part Ib: 36 evaluable patients with advanced colorectal cancer, 12 patients with head and neck squamous cell carcinoma and 12 patients with nasopharyngeal carcinoma.

7.2 Study site

Part Ia: Single center: Sun Yat-sen University Cancer Center

Part Ib: Multicenter: The leading site of the study is Sun Yat-sen University Cancer Center in Guangzhou. An additional 4-8 additional sites will be selected based on the progress of the trial.

7.3 Inclusion criteria

1) Gender: Male or female;
2) Age: ≥ 18 years, ≤ 75 years;
3) Life expectancy ≥ 12 weeks;
4) Phase Ia dose escalation phase: patients with histologically or cytologically confirmed locally advanced or metastatic solid tumors who have failed or cannot obtain standard treatment;
5) Phase Ib dose expansion phase: patients with histologically or cytologically confirmed locally advanced or metastatic colorectal cancer, head and neck squamous cell carcinoma or nasopharyngeal carcinoma, who have failed standard treatment, cannot tolerate standard treatment or cannot obtain standard treatment;
6) For patients participating in phase Ib clinical trial, their pathological samples shall be positive for EGFR expression by immunohistochemical detection;
7) Patients must have at least one measurable lesion as defined by RECIST v1.1;
8) ECOG score of 0-1;
9) Patient has organ function meeting the following laboratory test results at baseline examination at the time of enrollment:

Blood function

- Absolute neutrophil count ≥ 1.5 × 109/L;
- Platelets ≥ 100 × 109/L;
- Hemoglobin ≥ 9 g/dL;

Hepatic and renal function:

- Total bilirubin ≤ 1.5 × upper limit of normal (ULN);
- ALP ≤ 1.5 times ULN. If the patient has bone metastasis, ALP has no upper limit;

- AST and ALT: ≤ 2.5 times ULN for patients without liver metastasis or ≤ 3 times ULN for patients with liver metastasis;
- International normalized ratio < 1.5 times ULN (anticoagulant naïve) or international normalized ratio < 3 times ULN (anticoagulant naïve);
- Serum creatinine ≤ 1.5 times ULN;

10) Women of childbearing age must have a negative serum pregnancy test before entering this study and agree to take complete contraception from the beginning of the study to at least 6 months after administration of the study drug; female patients without childbearing potential should have spontaneous amenorrhea for at least 12 months; or have undergone oophorectomy, hysterectomy or tubal ligation more than 6 weeks before screening; in the case of unilateral oophorectomy, their fertility status will be determined by hormone levels;

11) Male patients must agree to use effective contraceptive measures (male condom with spermicide, or provide evidence of successful vasectomy; an intrauterine device with spermicide, female condom with spermicide, contraceptive sponge with spermicide, intravaginal contraceptive, diaphragm with spermicide, cervical cap with spermicide, or oral/implanted/or injectable contraceptives) from the start of the study to at least 6 months after study drug administration;

12) Patients voluntarily signed the written informed consent form to participate in this clinical trial, followed the study schedule and other requirements of the protocol.

7.4 Exclusion Criteria

Any subject who had any of the following conditions was excluded from the study

1) History of allergic reaction to any component of MRG003;

2) Patients with symptomatic central nervous system (CNS) metastases;

   Note: patients with a previous history of central nervous system metastases who have completed treatment can participate in this trial, that is, patients who have completed treatment for CNS metastases after radiotherapy or surgery 2 weeks before participating in this study and are neurologically stable (no new neurological deficits caused by brain metastases are found in clinical screening and no new lesions are found in central nervous system imaging) can participate in this study. If the patient requires steroids for treatment of CNS metastases, steroids must have been discontinued 2 weeks prior to the first dose;

3) Patients with a history of other primary malignancies, (except locally resected skin cancer and carcinoma in situ of the cervix, but melanoma needs to be excluded). Patients who previously suffered from other primary tumors but were

able to provide treatment for more than 3 years without tumor recurrence or progression can participate in this study;

4) Patients with a history of clinically significant liver disease, such as hepatitis C (positive hepatitis C antibody test) or chronic active hepatitis B (positive HBsAg for more than 6 months, HBV DNA ≥ 2000 IU/ml, ALT ≥ 2 ULN and excluding hepatitis caused by drugs or other reasons), alcoholic hepatitis, non-alcoholic steatohepatitis, hepatectomy, cirrhosis, etc.;

5) Patients with known human immunodeficiency virus (HIV) infection;

6) History of the following ophthalmologic abnormalities, e.g.:

- Severe dry eye syndrome;
- Keratoconjunctivitis sicca;
- Severe exposure keratitis;
- Any other condition that may result in an increased risk of corneal epithelial damage;

7) Patient has any serious and/or uncontrolled disease or other condition that, in the opinion of the Investigator and Sponsor, may compromise the patient's participation in this study, as follows:

- Uncontrolled diseases, or participation in this study may affect the control of these diseases;
- History of interstitial pneumonia, radiation pneumonitis, severe chronic obstructive pulmonary disease, severe pulmonary insufficiency, symptomatic bronchospasm;
- Life-threatening autoimmune diseases and ischemic diseases;

8) Patients with impaired cardiac function or clinically significant cardiac disease, including any of the following:

- Heart rate-corrected baseline QT interval calculated by Fridericia's formula $>$ 450 msec, or congenital QT prolongation syndrome;
- Concomitant diseases that may prolong QT interval as assessed by the investigator, such as autonomic neuropathy (due to diabetes or Parkinson's disease), HIV infection, liver cirrhosis, uncontrolled hypothyroidism, or heart failure;
- History of severe uncontrolled arrhythmia;

- Patients having: myocardial infarction, unstable angina pectoris, coronary artery bypass grafting, congestive heart failure, cerebrovascular accident or transient ischemic attack within 3 months prior to the first dose;

9) Use of chemotherapy, biological therapy, immunotherapy, radiotherapy, other clinical studies and other anti-tumor treatment within 4 weeks prior to the first dose; or have not recovered from side effects of the above treatment (CTCAE v4.03 grade 2 or higher). If the half-life of the antineoplastic drug used by the patient is very short (such as fluorouracil), this patient can be appropriately relaxed, which may be allowed to take the drug 2 weeks later. However, this individual case needs to be communicated with and agreed by the sponsor;

10) 4. Major surgery or surgical treatment for any reason before the first dose;

11) The patient has a planned surgery during the study period, or the investigator considers the patient requires surgery;

12) History of severe skin diseases requiring interruption of previous EGFR-targeted therapy; or chronic skin diseases requiring oral or intravenous therapy as prescribed;

13) The patient has any other concomitant disease that may increase the risk of toxicity as considered by the investigator or sponsor;

14) Pregnant or lactating.

7.5 Subject Withdrawal Criteria

7.5.1 Investigator's decision to withdraw a subject

Withdrawal of a subject from the trial refers to that the enrolled subject suffers from conditions unsuitable for continuation of the trial during the trial. The investigator decides to withdraw the subject from the trial, and the investigator decides to withdraw the subject from the trial for diagnosis and treatment measures.

（1） Unequivocal disease progression;

（2） Subject died;

（3） The subjects with adverse events and serious adverse events who are not suitable for continuation of the trial as judged by the investigator should continue to be followed up for 21 days or until the adverse reactions are relieved or recovered;

（4） Use of other therapies or drugs that affect the efficacy and safety judgment;

（5） Patients with poor medication compliance who cannot make a visit on time during two consecutive visit time windows.

7.5.2 Withdrawal by subject

According to the Good Clinical Practice (GCP), subjects have the right to withdraw from the study at any time for any reason, or they are lost to follow-up due to failure to observe and test the investigational drug but do not explicitly propose to withdraw from the study, which also belongs to "withdrawal" or "dropout". For subjects withdrawing from the trial, the reason for withdrawal should be known as much as possible and recorded in the eCRF. Possible reasons: withdrawal of the ICF; perceived poor efficacy; intolerance to some adverse reactions; inability to continue the clinical study due to work or life effects; loss to follow-up due to economic factors or unexplained reasons.

Regardless of the reasons, for the subjects who withdraw from the trial, the records of their last subject visit/follow-up cases (including laboratory tests and test indicators) should be retained, and the efficacy and adverse reactions should be analyzed in the full data set by taking them as the last test results and transferring them into the final results.

7.6 Criteria for Discontinuation

Trial suspension refers to stopping the trial halfway when the clinical trial has not been completed as per the protocol. The purpose of trial suspension is to protect the rights and interests of the subjects, ensure the quality of the trial and avoid unnecessary economic losses. The investigator should carefully document the reason for discontinuation. Criteria for discontinuation:

（1） Multiple serious adverse events occurred in the study, which affected the investigators' judgment on the safety of the investigational drug;

（2） The study found that the clinical trial protocol had major defects, making it difficult to evaluate the drug effect, especially affecting the safety and health of subjects;

（3） The investigators are found to have serious deviations in the implementation of study protocol and standard operating procedures (SOPs) during the implementation of the study, which cannot achieve the purpose of study evaluation;

（4） Significant management defects of the study or risks of the study are found in the IEC follow-up ethics review process, which require suspension of the ongoing study;

（5） The sponsor requests suspension (e.g., economic reasons, administrative reasons and/or the study effect fails to achieve the expected results);

（6） China Food and Drug Administration ordered to terminate the trial for some reason.

7.7 Exclusion criteria

（1） Accidental case;

（2） Those who meet the inclusion criteria and have not taken the drug after inclusion;

（3） The combination of drugs beyond the specified range, especially the combination of drugs, has a great impact on the evaluation of trial results and affects the efficacy and safety judgment;

（4） Participating in other clinical trials during the trial;

The reasons for exclusion should be explained, and the eCRF should be retained for future reference. The excluded cases were not included in the statistical analysis of efficacy; however, the excluded cases who had received the study drug treatment and had safety records could be included in the safety analysis as appropriate.

7.8 Criteria for Drop-out Cases

（1） Subjects withdraw by themselves (conscious efficacy is too poor, adverse reactions, etc.);

（2） Lost to follow-up;

（3） The investigator advised her to withdraw (poor compliance, serious comorbidities or complications, serious adverse events).

The reasons for dropout should be recorded in detail, and the last primary efficacy test result should be transferred to the final result for statistical analysis. The eCRF should be retained for future reference.

8 STUDY DRUG

8.1 Information about the study drug

8.1.1 Study Drug Shipment and Transfer

The product was manufactured and packaged under GMP conditions and stored and transported at 2-8°C in the dark. The drug will be provided by the sponsor and handed over to CRO, which will be responsible for the storage and transportation of the drug.

8.1.2 Study Drug Ingredients, Appearance, Packaging, and Labeling

The pharmaceutical components of MRG003 for injection include MRG003. Its active ingredient is recombinant humanized anti-EGFR monoclonal antibody-vcMMAE conjugate (MRG003).

MRG003 drug product is supplied as a white to off-white loose mass in a single-use vial with the label claim of 20 mg/vial and packaged as a box of 1 vial. It is a colorless to slightly yellow, clear to slightly opalescent solution after dissolution for intravenous drip. The MRG003 concentration after dissolution is 4 mg/mL.

8.1.3 Study Drug Storage and Stability Testing

The product should be stored and transported at 2-8°C, protected from light.

Shelf life of reconstituted solution

The product is stable for 24 hours at room temperature and 2 ~ 8°C after dissolution with sterile water for injection. The solution remaining after 24 hours should be discarded.

Validity period of the solution for infusion containing the reconstituted solution

The reconstituted MRG003 solution was diluted with 0.9% sodium chloride in normal saline to the drug concentration of 0.2-3.0 mg/mL, and was stable for 24 hours at room temperature. Since the diluted MRG003 solution does not contain preservatives, the prepared solution should be used as soon as possible, and the remaining solution after more than 24 hours should be discarded.

Do not use after the expiration date shown on this product.

8.1.4 Study Drug Preparation

Each vial is reconstituted with 5 mL of Sterile Water for Injection before use and is a colorless to slightly yellow, clear to slightly opalescent solution, free of visible particles.

Prior to administration, withdraw the appropriate volume of MRG003 reconstituted solution and slowly inject into a flexible bag or bottle of 0.9% Sodium Chloride Injection and gently invert several times to thoroughly mix the solution to avoid excessive foam

during mixing. The mixed solution is intravenously infused into the patient's body for 30 minutes to 3 hours.

8.1.5 Dose and Schedule

According to the actual body weight of the subjects, administration was designed according to the dose in this clinical protocol.

8.1.6 Administration method and administration period

MRG003 will be administered as an intravenous infusion on Day 1 of each cycle.

Each MRG003 dosing cycle is every 3 weeks (Q3W). Each subject received up to 8 dosing cycles throughout the study.

Prior to the start of each dosing cycle, subjects must meet the following conditions to receive intravenous infusion of MRG003:

- Neutrophil count $\geq 1.5 \times 10^9$/L and platelet count $\geq 75 \times 10^9$/L. Supportive therapy was allowed to improve the subject's hematologic condition (except prior to the first dose).
- Adverse reactions related to study drug must have improved to baseline or CTCAE v4.03 grade ≤ 1. For adverse reactions that do not result in fatal organ damage, such as hair loss, the investigator may decide whether the drug can be administered to the subject.

8.2 Dose Modifications, Modifications, and Delays

The investigator may delay the administration of study drug due to toxicity of study drug. Once a dose delay occurs, the scheduled time for subsequent doses should be adjusted later. However, if a subject delayed dosing for > 3 weeks (1 dosing cycle), the subject was considered withdrawn from the trial.

The investigator may also adjust the dose of study drug because of study drug toxicity, and a maximum of 2 dose reductions is allowed. However, patients in the initial dose 0.1 mg/kg group were not allowed to reduce the dose during the experiment, and patients in the second dose 0.3 mg/kg group were only allowed to reduce the dose once to 0.1 mg/kg during the experiment. If the subject is still intolerable after dose reduction for 2 times, the drug should be permanently discontinued and the patient should withdraw from the trial. If a subject's dose is reduced, subsequent doses will be adjusted according to the new dose, and re-escalation is not allowed.

Table 5: Dose Modification Table

Dose Level	MRG003 Dose

- 1	Previous Dose
- 2	First two doses
• If a subject delayed dosing for > 3 weeks, the subject was considered withdrawn from the trial<br>• If a subject required a dose reduction > -2 dose levels, the subject was permanently discontinued from the trial	

A subject may require a dose reduction for any of the following adverse events:

- For CTCAE v4.03 grade 3 to 4 non-hematologic adverse events related to drug toxicity that cannot be resolved to grade 2 or less within 72 hours after supportive care, study drug dose reduction is required. However, the investigator can determine to what extent the dose of the study drug can be not adjusted for adverse reactions that will not cause damage to vital organs, such as alopecia, which is recovered after supportive treatment;
- Grade 4 neutropenia lasting > 5 days;
- Febrile neutropenia;
- Grade 4 thrombocytopenia;
- Grade 2 irreversible peripheral sensory nerve abnormalities; Grade 2 or higher neurological diseases, which still fail to return to the baseline level within 4 weeks after administration after supportive treatment;
- Other adverse events decided by the investigator;

8.3 Management and Responsibilities for Study Drug

The investigator, the hospital pharmacist, or other personnel approved to store and dispense the study drug should ensure that the study drug is safely stored in the clinical study in strict accordance with the sponsor's instructions and applicable regulations, etc.

All study drugs must be dispensed according to the investigator's prescription and it is the investigator's responsibility to ensure that dispensing and return of study drugs are accurately recorded.

The sponsor must be notified immediately of any quality problems (such as failure of study drug storage conditions to meet the standards, appearance damage, missing attached documents, illegible label and expired shelf life) during the receipt and use of the study drug, and relevant reporting procedures will be initiated by the sponsor.

In no event shall the Investigator give the Study Drug to a third party, use the Study Drug for a Study subject as directly stated in the Non-Clinical Study Protocol, or otherwise dispose of the Study Drug.

8.4 Return and/or Destruction of Study Drug

8.4.1 Partially used or unused study drug

It is the responsibility of the sponsor and the clinical investigator to ensure destruction of all unused study drug. A detailed study drug return log was established by the investigator (or pharmacist) and signed by both the investigator and the monitor.

Considering the toxicity of the study drug, the used or partially used solution must be destroyed according to the usual procedures of each study site.

8.4.2 Possible recall

The sponsor may initiate the recall procedure due to the quality problem of the investigational product. At this time, it is the responsibility of the investigator to respond expeditiously to any request made by the sponsor to recall the study product and eliminate any potential hazards.

9 TRIAL PROCEDURES/VISIT SCHEDULE

9.1 Visit Schedule

Table 6: MRG003 Study Drug Phase I Clinical Trial Visit Schedule

	**Pre-screening period**	**Screening Period**	**Treatment period**													**Postdose Assessments**	**Efficacy and Survival Follow-up[a]**	
**Visit Item**	**Pre-screening**	**Screening**	**Cycle 1**								**Cycle 2[x]**				**Cycle 3 and subsequent cycles[x]**	**After administration**	**Every 6 weeks**	**Every 12 weeks**
**Scheduled Time Node (days)**	D-42	D-28[c]	D1[d]	D2	D3	D4	D5l	D8	D11	D15	D1	D2	D8	D15	D1	D21		
**± Visit Window (days)**							1	1	1	1		1	1	1	1	7	7	7
Biomarker Screening Informed Consent Form	X																	
Informed Consent Form for Clinical Study		X																
Inclusion and exclusion criteria		X	X															
Radiographi		X														X	X	

	Pre-screening period	Screening Period	Treatment period													Postdose Assessments	Efficacy and Survival Follow-up [a]	
**Visit Item**	**Pre-screening**	**Screening**	**Cycle 1**								**Cycle 2[x]**				**Cycle 3 and subsequent cycles[x]**	**After administration**	**Every 6 weeks**	**Every 12 weeks**
**Scheduled Time Node (days)**	D-42	D-28[c]	D1[d]	D2	D3	D4	D5l	D8	D11	D15	D1	D2	D8	D15	D1	D21		
c CT or MRI assessment[e]																		
Demographics		X																
Past medical history [f]		X																
Height and weight [w]		X	X								X				X	X		
Concomitant medication		X	Continuous recording															
Vital Signs [g]		X	X	X		X		X		X	X	X	X	X	X	X		
Physical Exam s		X	X					X		X	X		X	X	X	X		
Ophthalmic Examination [t]		X																
Performance status (ECOG score)		X	X								X				X	X		
	Project inspection																	
12 ECG		X	X								X				X	X		

	**Pre-screening period**	**Screening Period**	**Treatment period**													**Postdose Assessments**	**Efficacy and Survival Follow-up** [a]	
**Visit Item**	**Pre-screening**	**Screening**	**Cycle 1**								**Cycle 2**[x]				**Cycle 3 and subsequent cycles**[x]	**After administration**	**Every 6 weeks**	**Every 12 weeks**
**Scheduled Time Node (days)**	D-42	D-28[c]	D1[d]	D2	D3	D4	D5l	D8	D11	D15	D1	D2	D8	D15	D1	D21		
Blood routine [h]		X	X			X		X		X	X		X	X	X	X		
Coagulation function		X	X								X				X			
Blood chemistry [v]		X	X					X		X	X				X	X		
Urinalysis		X	X								X				X	X		
Serum pregnancy test [i]		X	X													Xi		
AIDS and hepatitis B and C testing [j]		X																
	Dosing																	
Intravenous drip of study drug [k]			X								X				X			
	PK, immunogenicity																	
MRG003 PK bleeding[l]			X	X	X	X	Xl	X	X	X	X	X	X	X	X	Xm		
Immunogenicity [n]			X								X				X	X		

	Pre-screening period	Screening Period	Treatment period													Postdose Assessments	Efficacy and Survival Follow-up [a]	
**Visit Item**	**Pre-screening**	**Screening**	**Cycle 1**								**Cycle 2**[x]				**Cycle 3 and subsequent cycles**[x]	**After administration**	**Every 6 weeks**	**Every 12 weeks**
**Scheduled Time Node (days)**	D-42	D-28[c]	D1[d]	D2	D3	D4	D5l	D8	D11	D15	D1	D2	D8	D15	D1	D21		
	Biomarkers																	
Tumor tissue sample [o]	X	X																
Carcinoembryonic antigen CEA[u]			X												X			
	ADVERSE REACTIONS																	
AEs and SAEs			Recording of AEs and SAEs															
	Efficacy Assessments																	
MTD p			Documentation of a DLT															
CR, PR, SD[q]																X	X	
PD																X	X	
PFS, DORr																X	X	
OS[r]			Continuously observe the survival condition of subjects															

(a) No PD was found in the subjects after the 21-day safety follow-up period after the end of administration, and the subjects should be followed up every 6 weeks for efficacy evaluation by imaging. After PD or other anti-tumor therapy, survival follow-up (via telephone) should be performed every 12 weeks until death.

(b) A total of 8 dosing cycles. From Cycle 3 (including Cycle 3), subsequent treatment cycles will be performed according to the procedures of Cycle 3, but the efficacy assessment will be performed every 6 weeks.

(c) The screening period is 4 weeks (28 days). Subjects in Phase Ib should enter the screening period after EGFR positive is confirmed by the central laboratory. One repeat screening is allowed for this study, and the possibility of a subject being re-screened will be discussed between the medical monitor and the sponsor.

	Pre-screening period	Screening Period	Treatment period													Postdose Assessments	Efficacy and Survival Follow-up[a]	
**Visit Item**	**Pre-screening**	**Screening**	**Cycle 1**								**Cycle 2[x]**				**Cycle 3 and subsequent cycles[x]**	**After administration**	**Every 6 weeks**	**Every 12 weeks**
**Scheduled Time Node (days)**	D-42	D-28[c]	D1[d]	D2	D3	D4	D5l	D8	D11	D15	D1	D2	D8	D15	D1	D21		

(d) See Section 9.3 for detailed operating procedures for the first dose.

The subjects generally arrive at the study site on the day before administration (D1 day) (D-1 day). The investigator may perform relevant examinations on the patient on Day -1 according to the actual situation of the patient. The same examinations may not be repeated on Day 1 after examination on Day -1, unless the investigator considers it necessary to perform re-examination.

If the interval between the date of screening examination and Day -1 is within 3 days, the investigator may consider not performing repeated laboratory tests on Day -1 and screening (the repeated tests may include: weight and height, serum pregnancy test, hematology and coagulation function test, blood biochemistry and urinalysis) according to the specific circumstances of subjects.

(e) Both CT and MRI can be used to assess tumor response. However, for each subject, the assessment method should remain consistent throughout the trial. At baseline, all patients with solid tumors must undergo CT/MRI scans of the chest, abdomen and pelvis according to RECIST v1.1 criteria. Patients with bone lesions should have a confirmatory bone scan at baseline. Tumour evaluation was to be performed by CT/MRI/radiology every 2 cycles (e.g., at the end of all even cycles). Patients with radiographically confirmed and documented tumor lesions at baseline will have a repeat radiologic examination every 2 cycles (i.e., at the end of each even cycle, or within 7 days prior to the start of Day 1 of each odd cycle) and at the End of Treatment/Early Termination Visit (except radiologic examinations completed within 28 days of the last assessment).

If the subject has undergone CT or MRI examination 4 times before the first dose and meets the requirements of this study, the investigator may consider it to be not necessary to repeat imaging examination during screening according to the specific circumstances of the subject.

(f) Past medical history includes previous history of bad habits such as smoking and alcohol abuse, previous history of disease, previous history of medical treatment/surgery, and history of anti-tumor therapy.

(g) Vital signs include: pulse, blood pressure and body temperature.

(h) See Section 10.2.1 for the specific description of laboratory tests, which should be performed before administration in each cycle to confirm whether the subject is eligible for administration.

(i) Serum pregnancy testing was performed on female subjects throughout the study and 21 after the dosing period.

(j) Includes: HIV (anti-HIV antibody) testing and hepatitis B virus (HBsAg) and hepatitis C virus antibody (anti-HCV antibody) testing.

(k) For MRG003 intravenous drip, see Section 9.3.

(l) Detailed plan for PK blood sampling 7-1, 7-2 and Table 8. Of these, Day 5 only has visit schedule in Phase Ia.

(m) Disposable blood sampling for PK and ADA measurements at the Day 21 visit after the end of dosing.

(n) Blood anti-therapeutic drug antibody (ADA) concentrations were measured in all subjects. In addition to the above table, ADA concentrations will also be tested before the start of the follow-up period at the end of the dosing period, see Table 7.

(o) Tumor tissue samples are used for biomarker detection and genetic testing of subjects.

(p) See Section 6.3.4 for evaluation of MTD.

(q) Subjects will be evaluated for tumor response every 6 weeks (at the end of the double dose cycle, i.e., before the start of the next dose cycle), and if a subject experiences a CR or PR, confirmation by the same method should be performed 6 weeks later.

(r) For efficacy assessments of PFS, DOR, and OS, see Section 6.5.

(s) Physical examination included head and neck, skin, lymph nodes, thyroid, musculoskeletal, extremities, cardiovascular, respiratory, abdominal, and neurological systems, and was performed only on the first day of the

	Pre-screening period	Screening Period	Treatment period													Postdose Assessments	Efficacy and Survival Follow-up[a]	
**Visit Item**	**Pre-screening**	**Screening**	**Cycle 1**								**Cycle 2[x]**				**Cycle 3 and subsequent cycles[x]**	**After administration**	**Every 6 weeks**	**Every 12 weeks**
**Scheduled Time Node (days)**	D-42	D-28[c]	D1[d]	D2	D3	D4	D5l	D8	D11	D15	D1	D2	D8	D15	D1	D21		

cycle after Cycle 3. Photographs can be taken and archived locally on the skin of representative lesions.

(t) Ophthalmic examination Ocular examination, including visual acuity, slit-lamp examination and dry eye examination such as tear secretion test, is only performed by ophthalmologists during the screening period and when ocular adverse reactions occur as needed.

(u) Blood samples for carcinoembryonic antigen were collected from all colorectal patients before administration on the first day of each odd cycle (1, 3, 5, 7).

(v) If the subject has grade 2 or higher infusion reaction, additional blood may be drawn for the detection of complement C3a, C4, CH50 and serum tryptase.

(w) Body weight may be checked prior to dosing within 3 days prior to dosing in each dosing cycle. Except for Cycle 1, the height test for Cycles 2-8 may not be performed.

(x) In Cycles 2 to 8, the patient may be hospitalized one day in advance. After the patient is admitted to the hospital, the investigator arranges the patient to have various examinations on the day of dosing in the table on the day before dosing. However, for dosing and PK, ADA blood sampling should be performed on the day of D1.

983

9.2 PRESCREENING (APPLICABLE FOR PHASE IB)

Patients must have EGFR expression confirmed by central laboratory within 42 days prior to the first dose of study drug. Details regarding collection, storage, shipping, and analysis will be provided in the Laboratory Manual.

During pre-screening, the following criteria are prerequisite for central confirmation of EGFR expression:

- Patients with histologically or cytologically confirmed locally advanced or metastatic colorectal cancer, head and neck squamous cell carcinoma or nasopharyngeal carcinoma;
- The information about the tumor pathological sections (5 sections) used for detecting the biomarker EGFR must be provided;
- Written informed consent must be obtained from the patient for collection, storage, and analysis of the tissue slides/paraffin blocks in accordance with International Conference on Harmonisation ICH-GCP and Chinese GCP regulations.

9.3 Screening Period (Day -28 to Day -1)

1) Patients should sign the informed consent form before initiation of any substantial trial procedures;
2) Establish a patient screening form to record the subject's initials, date of birth, gender, ethnicity and contact information;
3) Demographic data include gender, ethnicity and date of birth;
4) Past medical history includes history of smoking, alcohol abuse and other bad habits, past medical history, past medical treatment/surgery history, and history of tumor diagnosis;
5) History of concomitant medication and anti-tumor treatment;
6) Physical examination: including head and neck, skin, lymph nodes, thyroid, musculoskeletal, limb, cardiovascular, respiratory, abdominal and neurological systems and performance status (using ECOG score);
7) Ophthalmic examination: including visual acuity, slit-lamp examination and examination of dry eye such as tear secretion test.
8) Height and weight
9) Vital signs (pulse/blood pressure/temperature);

10) Radiographic assessment: Both CT and MRI can be used to evaluate tumor response. However, for each subject, the assessment method should remain consistent throughout the trial period.

11) 12 Electrocardiogram;

12) Urine routine;

13) Hematology and coagulation function test (see Section 10.2.1 for details);

14) Blood biochemistry (see Section 10.2.1 for details);

15) Serum pregnancy test (in women of childbearing potential);

16) Detection of HIV (anti-HIV antibody), hepatitis B virus (HBsAg) and hepatitis C virus (anti-HCV antibody);

17) According to the inclusion and exclusion criteria, determine whether to use the patient;

18) The pathological tumor samples of subjects are collected for the detection of biomarker information, including the detection of EGFR protein expression (Phase Ia only), EGFR targeted sequencing, KRAS, NRAS and BRAF gene hotspot mutation.

9.4 First dosing period (Day -1 ~ 1)

9.4.1 Admission to Phase I Clinical Trials Unit

Eligible subjects were admitted to the Phase I Clinical Trials Unit (CRU) one day before MRG003 administration and were examined as follows:

1) Re-confirm that the subject meets the inclusion and exclusion criteria;

2) Physical examination: including performance status (using ECOG score);

3) Body weight and height;

4) Vital signs (pulse/blood pressure/temperature);

5) Symptoms and signs related to expected adverse reactions of study drugs: including pain and allergy; symptoms and signs of skin, digestive tract, cardiovascular, nervous, facial features, bones and other systems, and establish baseline levels of expected adverse reactions;

6) Serum pregnancy test (in women of childbearing potential);

7) 12 Electrocardiogram;

8) Blood routine and coagulation function test;

9) Blood biochemistry;

10) Urinalysis;

11) Carcinoembryonic antigen testing is required in all patients with colorectal cancer.

9.4.2 Before intravenous drip of MRG003

1) Collect 5 ml blood sample for PK and ADA analysis before intravenous drip of MRG003;

2) Vital signs: pulse, blood pressure and body temperature;

3) 12 Electrocardiogram.

4) Subjects may be preconditioned for possible allergic reactions.

9.4.3 First IV drip (Day 1)

1) The subject received intravenous infusion of MRG003 over 30 minutes to 3 hours. If a subject requires significant additional hydration, this should be recorded on the eCRF form.

2) To determine PK, anti-therapeutic antibody (ADA), and drug toxicity, blood sampling time points before and after intravenous infusion of MRG003 are provided in Tables 7-1 and 7-2. See Table 8 for test items, blood collection volume and treatment method. If the time of blood collection for other items overlaps with that of PK blood collection, non-anticoagulated blood samples for PK determination shall be collected preferentially.

3) Within 24 hours after the end of intravenous drip of MRG003, closely monitor the vital signs of subjects;

4) Within 4 hours after the end of intravenous drip of MRG003, measure the ECG of subject 12;

5) Pay close attention to the abnormal changes in symptoms and signs of subjects that may be caused by drug toxicity within 24 hours after intravenous drip of MRG003, and record the adverse reactions or serious adverse reactions occurred;

6) After intravenous drip of MRG003, the concomitant medication of the subject was recorded in detail.

Table 7-1: Phase Ia PK, Immunogenicity Blood Sampling Points, and Vital Sign Observation Time Points

Cycle	Days	Time Location	Blood sampling time point (hr)	Blood sampling time range	Epilepsy	Immunogenicity	Vital Signs Observed

1st Cycle	1	Before instillation	0b	- 1 hr	√	√ c	√
		Start of infusion	10 min after start	± 5 min	√		√
		End of infusion	0c	+ 10 min	√		√
			2	± 10 min	√		√
			4		√		
			8		√		
			12		√		
			18		√		
	2	End of infusion	24	± 2 hr	√		√
	3[a]		48		√		
	4[a]		72		√		√
	5[a]		96		√		
	8		168		√		√
	1I[a]		240		√		
	15		336		√		√
Cycle 2	1	Before instillation	0[b]	- 1 hr	√	√ c	√
		End of infusion	0[c]	+ 10 min	√		√
			4	± 10 min	√		√
	2	End of infusion	24	± 2 hr	√		√
	8		168		√		√
	15		336		√		√
Subsequent Dosing Cycles	1	Before instillation	0[b]	- 1 hr	√	√ c	√
		End of infusion	0[c]	+ 10 min	√		√
		Follow-up period (21 days after the end of the last administration)			√	√	√

A. PK on days 3, 4, 5, and 11 blood sample blood sampling points are only applicable to the first dosing cycle of the dose escalation phase.

B. blood samples must be collected prior to instillation.

C. See drug preparation guidelines for infusion time. The end point of infusion was recorded as 0 hour.

D. completed within 10 minutes before and after blood collection, and if the patient is scheduled for follow-up in the outpatient clinic at a later stage, the time window can be extended to within 30 minutes before and after PK blood collection.

Table 7-2: Phase Ib PK, Immunogenicity Blood Sampling Points, and Vital Sign Observation Time Points

Cycle	Days	Time Location	Blood sampling time point (hr)	Blood sampling time range	Epilepsy	Immunogenicity	Vital Signs Observation d
1st Cycle	1	Before instillation	0b	- 1 hr	√	√ c	√
		End of infusion	0c	+ 10 min	√		√
			4	± 10 min	√		
			12		√		
	2	End of infusion	24	± 2 hr	√		√
	3[a]		48		√		
	4[a]		72		√		√
	8		168		√		√
	1I[a]		240		√		
	15		336		√		√
Cycle 2	1	Before instillation	0[b]	- 1 hr	√	√ c	√
		End of infusion	0[c]	+ 10 min	√		√
			4	± 10 min	√		√
	2	End of infusion	24	± 2 hr	√		√
	8		168		√		√
	15		336		√		√

Subsequent Dosing Cycles	1	Before instillation	0[b]	- 1 hr	√	√ [c]	√
		End of infusion	0[c]	+ 10 min	√		√
		Follow-up period (21 days after the end of the last administration)			√	√	√

A. PK at 3, 4, and 11 day blood sampling points is only suitable for the first dosing cycle of the dose expansion phase.

B. blood samples must be collected prior to instillation.

C. See drug preparation guidelines for infusion time. The end point of infusion was recorded as 0 hour.

D. completed within 10 minutes before and after blood collection, and if the patient is scheduled for follow-up in the outpatient clinic at a later stage, the time window can be extended to within 30 minutes before and after PK blood collection.

Table 8: Test Items and Blood Volume

	**Test item**	**Drug Ingredient**	**Serum**	**Plasma**	**Quail [a]**	**Blood Volume**
Pharmacology	Total Antibody	TAb	√			5 mL
	Conjugated Antibody	ADC	√			
	Free Drug	MMAE	√			
Immunogenicity	Anti-therapeutic drug antibody	ADA	√			3.5 mL
Blood routine [a]					√	1 mL
Blood biochemistry			√			3 mL
Coagulation test [b]					√	2 mL

A EDTA dipotassium salt anticoagulant vacuum blood collection tube;
B Sodium citrate anticoagulant vacuum blood collection tube;

9.5 Ia Treatment Period and Post-Treatment Assessments

1) Subjects will receive intravenous infusion of MRG003 at different doses, and if the subject does not experience DLT during the dose escalation period (the first dosing cycle), the administration should be continued in the second cycle. Tumor assessments were also performed every 6 weeks. And may receive a third cycle and subsequent cycles at this dose for up to 8 cycles. Eight dosing cycles will take longer than 24 weeks, with a maximum of approximately 15 visits. One subject received only one dose of the tolerability trial.

2) Refer to Section 9.3 for the operation of first dosing.

3) Observe and record DLTs and determine MTD or RP2D dose in Cycle 1 only.

4) Blood samples were collected from all subjects during the dosing period for measurement of PK parameters. See Table 7-1.

5) Blood will be drawn to measure the subject's ADA antibody concentration at specified visits (see Table 6 of the visit schedule) to assess immunogenicity of the study drug.

6) Subjects were assessed for antitumor efficacy every 6 weeks. Efficacy assessment methods are described in Section 6.5.

7) Patients may receive up to 8 cycles of study drug. At the end of the last dosing, the study site should recall the subjects to the study site about 21 days later for a safety visit to understand the resolution of ongoing adverse events or abnormal changes in laboratory tests, and to evaluate the new adverse events, serious adverse events, new drugs or changes in current medication that have occurred since the last assessment. Any new occurrence that occurs during the safety follow-up period needs to be recorded in the eCRF as appropriate.

8) Vital sign parameters (pulse, blood pressure, body temperature), ECG, physical examination, including performance status score, height and weight, hematology, coagulation, blood biochemistry and urinalysis will be performed during the visits specified in Table 6. In addition, attention should be paid to whether additional examinations are required due to clinical indications at each visit during the treatment period, such as vital signs, ECG, neurological examination, blood routine examination, coagulation function test, blood biochemistry test, urinalysis, pregnancy test, etc. In addition, carcinoembryonic antigen test should be performed in odd dosing cycles (i.e., Cycle 1, Cycle 3, Cycle 5 and Cycle 7) for all patients with colorectal cancer.

9) Record AEs/SAEs and concomitant medications of subjects.

10) Subject survival status was recorded.

9.6 Ib Treatment Period and Post-Treatment Assessments

1) Subjects will receive MRG003 at MTD/RP2D via intravenous infusion for 8 cycles; 8 cycles will take more than 24 weeks for a total of 15 visits.

2) Refer to Section 9.2 for the operations during the screening period and Section 9.3 for the operations during the first dosing.

3) The dosing cycle was performed according to 6.

4) Blood samples will be collected during the dosing period to determine PK parameters as specified in Table 7-2.

5) Blood will be drawn to measure the subject's ADA antibody concentration at specified visits (see Table 6 of the visit schedule) to assess immunogenicity of the study drug.

6) Subjects were assessed for antitumor efficacy every 6 weeks. Subjects with response of CR and PR should be assessed for response confirmation at subsequent Week 6. Efficacy assessment methods are described in Section 6.5. In addition to ORR indicators for anti-tumor efficacy, PFS, OS and DOR indicators for anti-tumor efficacy will also be evaluated.

7) At the end of the last dosing, the study site should recall the subjects to the study site about 21 days later for a safety visit to understand the resolution of ongoing adverse events or abnormal changes in laboratory tests, and to evaluate the new adverse events, serious adverse events, new drugs or changes in current medication that have occurred since the last assessment.

8) Vital sign parameters (pulse, blood pressure, body temperature), ECG, physical examination, including performance status score, height and weight, hematology, coagulation, blood biochemistry and urinalysis should be performed during the visits specified in Table 6. In addition, attention should be paid to whether additional examinations are required due to clinical indications at each visit during treatment, such as vital signs, ECG, neurological examination, routine blood examination, coagulation function test, blood biochemistry, urinalysis, and serum pregnancy test.

9) Record AEs/SAEs and concomitant medications of subjects.

10) Subject survival was recorded.

9.7 Efficacy and Survival Follow-up

For subjects without disease progression after the end of 21-day treatment visit, anti-tumor efficacy assessment and survival follow-up should be performed every 6 weeks until disease progression or other anti-tumor treatment. After confirmation of disease progression or receipt of other antineoplastic therapy, patients were followed for survival every 12 weeks until death or study termination. See the data management plan of this study for the cut-off point of data collection in the case report form.

9.8 Early termination of treatment

Treatment of a subject may be discontinued early in the following circumstances:

- In case of uncontrolled adverse events, the investigator decides to stop treatment;
- The subject experienced disease progression;
- Subject died;
- Withdrawal of informed consent by the subject;
- Subject lost to follow-up.

In any case of premature treatment discontinuation, the investigator should make every effort to inquire about the reason for premature treatment discontinuation and record the discontinuation on the subject's eCRF form. When a subject prematurely discontinues treatment due to an AE/SAE, the investigator should follow the subject until the condition is cured or stable, and continue to complete imaging assessments and survival follow-up.

10 TEST OPERATION GUIDELINE

10.1 General operation

- Physical examination and baseline indicators:

  Standard demographic and baseline characteristics (including age, gender, race, height and weight, etc.), medical history, cancer diagnosis and prior anticancer therapy will be collected at baseline.

- Imaging examination:

  CT, MRI: CT is currently the best and reproducible test for assessing anti-tumor efficacy. The lesion measurements defined in this study according to RECIST v1.1 were based on the assumption that the CT scan slice thickness did not exceed 5 mm. When CT scans have slice thickness greater than 5 mm, the minimum size for a measurable lesion (diameter) should be twice the slice thickness. MRI may also be used in certain situations (e.g. for body scans). For each identified and reported lesion, the same measurement technique and assessment method should be used at baseline and at follow-up. Further details on the use of CT and MRI examinations to assess objective tumour response are provided in Appendix II.

10.2 Laboratory assessments

10.2.1 Clinical Laboratory Assessments

10.2.1.1 Blood routine examination

The subject's examination includes: red blood cell, white blood cell (neutrophil, eosinophil, basophil, lymphocyte, monocyte), hemoglobin, platelet and other related tests (white blood cell differential and absolute count collected and recorded).

10.2.1.2 Coagulation test

Coagulation test for subjects includes: prothrombin time (PT), activated partial thromboplastin time (APTT), fibrinogen, international normalized ratio (INR) and other related tests.

10.2.1.3 Blood biochemistry

The subject's serum biochemical examination (SBP) includes aspartate aminotransferase (AST), alanine aminotransferase (ALT), alkaline phosphatase, amylase, lipase, lactate dehydrogenase, γ-glutamyltransferase direct bilirubin and total bilirubin, total protein, blood glucose, potassium, sodium, calcium, creatinine, blood urea nitrogen (BUN) and other related tests.

If the serum creatinine concentration is > 1.5 × ULN, creatinine clearance will be calculated (calculated according to age using the following formula).

- Cockroft-Gault calculation formula (female):

  Endogenous creatinine clearance rate (mL/min) = [(140-age) × body weight * (kg) × 0.85]/[72 × serum creatinine (mg/dl)]

  OR [(140-age) × weight * (kg) × 0.85]/[0.818 × serum creatinine (umol/L)]

- Cockroft-Gault calculation formula (male):

  Endogenous creatinine clearance rate (mL/min) = [(140-age) × body weight * (kg)]/[72 × serum creatinine (mg/dl)]

  OR [(140-age) × weight * (kg)]/[0.818 × serum creatinine (umol/L)]

  * Body weight: Body weight on D1 was used to calculate all creatinine clearance in the first dosing cycle

10.2.1.4 Carcinoembryonic antigen test

Carcinoembryonic antigen testing was performed in all colorectal cancer patients during odd dosing cycles (i.e., Cycles 1, 3, 5, and 7).

10.2.1.5 Urine test

Urinalysis: urine color, transparency, pH, specific gravity, white blood cells, nitrite, occult blood, qualitative protein, qualitative and microscopic examination of urine glucose (if white blood cells, nitrite, total protein, or occult blood are positive, microscopic examination will be performed).

10.2.1.6 Serum pregnancy test

Serum human chorionic gonadotropin (HCG) levels were measured by conventional laboratory methods in women for the diagnosis of early pregnancy. Pregnant women generally have a positive serum pregnancy test after 35 days of menopause.

10.2.1.7 Infectious Disease Testing

To detect HIV, HBsAg and anti-HCV antibody in the blood of subjects.

10.2.2 Special Experimental Assessments

10.2.2.1 Genetic testing

Handling of new tissue biopsy specimens or archived specimens is described in the Laboratory Manual. Subject pathology specimens should be sent to a third party laboratory at pre-screening and/or screening as requested by the sponsor in an appropriate manner. Third-party laboratories are responsible for detecting EGFR protein expression and EGFR targeted sequencing, KRAS, NRAS and BRAF gene hotspot mutation detection.

10.2.2.2 Determination of metabolic parameters of study drug

Blood samples (8) were collected according to the visit schedule (Table 6 and Table 7), and the serum concentrations of three components of MRG003: conjugated antibody (MRG003), total antibody (conjugated antibody + naked antibody), and free small molecule toxin MMAE were determined by a validated method at a third-party bioanalytical laboratory.

In the first dosing cycle of Phase Ia and Phase Ib, PK parameters of MRG003 in all subjects will be analyzed by non-compartmental method, but not limited to C_{max}, T_{max}, $t_{1/2}$, MRT, V_d, CL, AUC_{0-t} and AUC_{0-inf}.

10.2.2.3 Study Drug Immunogenicity Assay

Immunogenicity refers to the drug attribute that protein drugs are injected into patients to cause adverse immune response. Immunogenicity can interfere with the kinetic parameters of protein drugs and the effectiveness of drugs. Anti-therapeutic drug antibodies (ADAs) have the potential to trigger allergic reactions, anaphylactic shock, and inflammatory reactions in patients.

In this study, blood was collected from subjects (see Table 6 and Table 7 for details) before the start of each dosing cycle and at the end of the dosing cycle (before the next dosing) to extract serum samples (8). Serum samples were temporarily stored at the laboratory of the research hospital below − 60 ° C. Subject serum samples will be shipped to a third party bioanalytical laboratory by appropriate methods on schedule at the request of the Sponsor. Immunogenicity and corresponding ADA assays for MRG003 included a screening assay, a confirmatory assay, and an assay.

10.3 Permitted concomitant medications and treatments

Due to the unique nature of oncology patients, subjects were allowed to receive adequate supportive care during the study as medically necessary and documented in the eCRF.

1) During the trial treatment, when the study drug causes acute toxic reactions, such as febrile neutropenia, the investigators should decide to give the subjects biological response modifiers (BRMs), such as G-CSF or GM-CSF, in combination with other concomitant symptoms and risk factors of clinical patients. The specific protocol can follow the clinical medication routine of each hospital; however, the use of biological response modifiers for patients to meet the trial inclusion criteria is not allowed, and the use of prophylactic biological response modifiers during the first dosing cycle is not allowed.
2) Prophylactic anti-allergic treatment, preventive antiemetic treatment;
3) Therapeutic use of antibiotics, e.g., in case of febrile neutropenia or infection;
4) In case of diarrhea, antidiarrheal treatment should be performed according to the routine of the hospital;

5) Palliative radiotherapy for analgesic purposes may be given (if bone metastases occur) to control pain. The irradiated area should be as small as possible. No target tumor lesion should be included in the irradiated area, otherwise the patient will be excluded from the trial, and the patient will not participate in the evaluation of tumor response. Radiation area can not be used as the parameter for response evaluation;
6) Other symptomatic treatment for drug toxicity may be given at the discretion of the investigator based on the specific condition of the subject and should be recorded in detail in the eCRF.

10.4 Prohibited concomitant medications and treatments

1) Patients are not allowed to receive other forms of anti-tumor therapy, including chemotherapy, immunotherapy, endocrine anti-tumor therapy, surgical therapy or other investigational drugs during the study. Whether the concomitant drug has anti-tumor effect shall be subject to the content in the package insert. Any disease progression requiring other anti-tumor treatment will lead to premature discontinuation of the study treatment;

2) Administration of radiotherapy (except palliative radiotherapy for pain relief purposes) is not allowed.

10.5 Post-Study Treatment

After the end or early termination of the study treatment, the investigator may decide to further treat the patient according to clinical practice. Subsequent anticancer therapy should also be recorded.

10.6 Treatment Accountability and Compliance

A pre-printed drug dispensing log and site drug accountability logs will be provided by the sponsor representative. Compliance with study treatment by the investigator is the responsibility of the investigator and is supervised by the sponsor representative through on-site monitoring visits. The investigator should maintain complete and accurate records of drug use data.

The person responsible for dispensing the drug recorded the use of all study drugs. Labels of MRG003 vials administered or dispensed to the patient must be complete (with the patient number and visit number, respectively). The production batch number and the expiry date of the investigational drug (MRG003 and sterile water for injection) must be recorded in the eCRF/drug accountability form, and the number of vials used and the administration date in each cycle must also be recorded (MRG003).

The person responsible for administration is responsible for recording the exact date and time that the patient was dosed. If there is an interruption in the infusion process, it should be recorded in the eCRF with the reason.

11 SAFETY ASSESSMENTS

In this study, all subjects who received intravenous infusion of MRG003 were evaluated for drug toxicity throughout.

11.1 Definition of different types of adverse events

11.1.1 Adverse Event Definitions

Definition of adverse event (AE): Any subject entering this trial after signing informed consent form develops or worsens any clinical condition, symptom, syndrome or disease affecting health during the clinical study, which is considered as an AE. Laboratory, vital sign, or ECG abnormalities were recorded as adverse events only if they were medically significant, i.e., symptomatic, requiring symptomatic treatment, leading to discontinuation, and/or fulfilling a seriousness criterion. An adverse event may be: a new disease; a worsening of symptoms or signs during treatment, or a worsening of a concomitant disease; an "adverse event" term does not imply a causal relationship to the trial drug.

In general, efficacy endpoints predefined in the protocol are not to be considered as AEs unless, due to the course, seriousness, or any other characteristics of such events, the investigator, in his/her opinion, considers these events to be exceptional in this medical setting, based on his/her best medical judgment.

11.1.2 Definition of Serious Adverse Event

A serious adverse event (SAE) is an adverse event that, at any dose of study drug, results in:

- Means a patient died; or
- Life-threatening; or
- Required or prolonged hospitalization; or
- Results in persistent or significant disability or incapacity; or
- Results in congenital anomaly or birth defect;
- Other medically important event:

Note: The term "life-threatening" in the definition of "serious adverse event" refers to an event in which the patient is at immediate risk of death from the event; it does not refer to an event that hypothetically might have caused death if it were more severe.

Hospitalization or prolongation of hospitalization will not be considered an adverse event if the following conditions are met: hospitalization planned before enrollment in the study; a mobile hospitalization (12 hours); a hospitalization that is part of the normal treatment or monitoring of the disease under study but is not caused by a worsening of the disease; a hospitalization that is only related

to socioeconomic factors and not related to the disease (e.g., hospitalization for medical insurance reimbursement, accommodation problems or physical examination needs).

11.2 Classification of adverse events

11.2.1 Severity of adverse event

All adverse events will be graded for severity according to the International System for Assessment of Adverse Drug Reactions to Cancer chemotherapy CTCAE v4.03 and refer to the details reported on the eCRF. In the event of any death, an appropriate death report form was completed. If an adverse event occurred that was not included in the NCI-CTCAE, it was graded using a quintile scale (mild, moderate, severe, life-threatening, death), 9.

Table 9: Adverse Event Severity Grading

Mild	Discomfort but no effect on normal daily activities
Moderate	Discomfort sufficient to reduce or affect daily activities
Severe	Inability to work or perform normal daily activities
Life Threatening	Immediately life-threatening
Death	Death related to AE

11.2.2 Relationship of adverse event to study drug

The investigator should determine the causality of adverse drug event/reaction and analyze the correlation. The analytical methods used mainly follow the following five principles (see Table 10).

Table 10: Relationship between adverse reactions and study drug

Serial number	Problem	Yes	None	Unknown
1	Whether there is a reasonable temporal relationship between medication and the occurrence of adverse reaction/event?	□	□	□
2	Whether the reaction conforms to the known type of	□	□	□

	adverse reaction of the drug?			
3	Did the reaction/event disappear or abate after stopping the drug or reducing the dose?	□	□	□
4	Did Reaction/Event Reappear After Reintroduction of Suspect Drug?	□	□	□
5	Can the reaction/event be explained by the effect of concomitant medication, progression of the patient's condition, effect of other therapies?	□	□	□

Relevance evaluation: According to the above five principles of adverse event/reaction analysis, the relevance evaluation is classified as positive, probable, possible, unlikely and unrelated (see Table 11).

Table 11: Relatedness of Adverse Events to Study Drug

	**Question 1**	**Question 2**	**Question 3**	**Question 4**	**Question 5**
Definitely related	+	+	+	+	-
Probably related	+	+	+	?	-
Possibly related	+	±	±?	?	±?
Unlikely related	-	-	±?	?	±?
Unrelated	-	-	-	?	+
Note: + indicates yes; - indicates no; ± indicates it is difficult to judge yes or no;? Indicates unknown;<br><br>Questions 1, 2, 3, 4, 5 10.					

The determination of correlation between adverse events and drug is the result of individual judgment by the investigator. When the principal investigator summarizes the clinical trial data and summarizes the safety data, the overall judgment shall be made according to the drug ingredients, dosage regimen, results of preclinical safety study, frequency, severity and trend of adverse events.

11.3 Frequency and duration of adverse events

The frequency and duration of adverse events should be recorded and reported in the eCRF.

11.4 Adverse event monitoring

The monitoring and reporting of adverse events is a necessary item in the daily work of clinical trials. All adverse events must be managed and reported in accordance with applicable regulations and applicable regulatory requirements and included in the final study report.

11.5 Recording and reporting of adverse events

11.5.1 AE recording

All adverse events, regardless of severity or relationship to the study product, shall be recorded in the EDC system from the signing of the informed consent form at the first visit scheduled in the clinical trial protocol, throughout the study treatment period and post-treatment evaluation period (within 21 days after the last administration of the study drug). Even if the subjects have completed the study treatment, they must be followed up until the adverse event is completely relieved or the event can be fully explained. The records shall include:

- Event name;
- Onset date and duration (start and stop times, or ongoing at visit);
- Severity (mild, moderate, severe or grade);
- Relationship to the study drug (definite, probable, possible, unlikely related, to be evaluated);
- Whether the investigational drug is stopped;
- AE action taken;
- Outcome (disappearance, remission, persistence, aggravation, death).

The investigator should take appropriate measures to ensure the health and safety of the subjects according to the AE; the specialized ward should ensure the supply of relevant rescue equipment and drugs to ensure timely treatment in case of emergency.

11.5.2 SAE Reporting

Any SAE occurring during the trial (whether or not related to the investigational product) should be reported to Shanghai Miracogen Inc., Covance PV & Drug Safety,

clinical study institution and its Ethics Committee by fax/email within 24 hours and reported to China Food and Drug Administration.

Contact information for SAE report:

Covance Pharmacovigilance and Drug Safety: SAEIntake@covance.com

All SAEs were to be followed up by the investigator until the final outcome was known. The investigator should fill in the SAE form for tracking, tick the "Follow-up Report" or "Final Report" column and fax/email it to Covance PV & Drug Safety, Clinical Trial Institution, Ethics Committee and China Food and Drug Administration. The frequency of follow-up was determined by the investigator on a case-by-case basis.

Note: For general AE events, medical and scientific judgment should be exercised in deciding whether expedited reporting is appropriate within 24 hours, such as important medical events that are not immediately life-threatening or result in death or hospitalization but may jeopardize the patient or may require intervention to prevent further outcomes in the definition of SAE. Some examples of such events are: treatment in a Phase I clinical trial ward, asymptomatic ALT elevations ≥ 10 × ULN due to allergic bronchospasm, cachexia, convulsions, or do not result in hospitalization, or development of drug dependency or drug abuse.

11.5.3 Drug-induced liver injury

Drug-induced liver injury is one of the most common and serious adverse drug reactions, and can cause acute liver failure or even death in severe cases. Hy's rule has important reference value for judging the prognosis of drug-induced liver injury. The core content is: in clinical trials, if there are subjects with serum ALT or AST ≥ 3 ULN and total bilirubin ≥ 2 ULN (not necessarily at the same time), it meets the potential Hy's law. If the subject meets potential Hy's Law and there is no other reason than the investigational product to explain the increased laboratory values. Alternative causes may include cholestasis, viral hepatitis, or other drug effects as indicated by elevated alkaline phosphatase (ALP). If the investigator identifies a potential Hy's Law case, the SAE Report Form should be completed and reported to the sponsor (or its designee) in an expedited manner within 24 hours. The investigator should then assess whether the case meets Hy's law and will report the results.

11.5.4 Serious Adverse Events Due to Disease Progression

Serious adverse events due to disease progression for all subjects treated with MRG003 should be reported according to the SAE reporting requirements within 21 days after signing the informed consent form at the first visit scheduled in the clinical trial protocol, throughout the study treatment period, and after the last dose of the study drug.

Serious adverse events due to disease progression that occur during the efficacy and survival follow-up periods of 21 months after the last dose of study drug do not need to be reported as SAEs.

11.5.5 Death

Any death, including death due to tumor progression, that occurs within 21 days of signing of informed consent and following the last cycle of study drug administration must be reported within 24 hours to a monitor appointed by the sponsor representative, regardless of the relationship between the death and the study drug. If a death occurs during the efficacy and survival follow-up periods, only those reports need to be considered serious if the relationship to study drug is deemed possible. All deaths should be recorded in the death report section of the eCRF, regardless of the causal relationship with the study drug.

11.5.6 Pregnancy

- In all cases, the pregnancy should be recorded and a pregnancy report should be completed.
- The Investigator should inform the patient (or legal representative) of the risks of continuing the pregnancy and the possible effects on the fetus.
- Female patients must be instructed that if they become pregnant during the study, they must discontinue any study medication and immediately report to the investigator. Pregnancies occurring within 90 days of completion of study drug must also be reported to the investigator. The investigator should report to the sponsor (or its designee) in an expedited manner within 24 hours.
- Any pregnancies in the partner of a male patient participating in the study should also be reported immediately to the investigator, who will also report in an expedited manner according to the procedures described above.
- The pregnancy must be followed up until the outcome is determined.

11.5.7 Overdose

In the event of an accidental or intentional overdose with the study drug (generally, an overdose is defined as at least twice the maximum daily dose per protocol), even if the seriousness criterion is not met, the Overdose Reporting Form should be completed and reported to the sponsor immediately (i.e., within 24 hours).

12 CLINICAL MONITORING

12.1 Investigator Responsibilities

The investigator undertakes to conduct this clinical trial in accordance with this study protocol, Good Clinical Practice (GCP) and applicable laws and regulations.

The investigator was required to ensure that the various procedures required by the clinical trial protocol and the various study procedures proposed by the sponsor, including safety regulations, were followed. The investigator agrees to provide reliable data and various information required by the clinical trial protocol in an accurate and legal manner in accordance with the instructions provided, and to ensure the sponsor's representative has direct access to/access to source documents.

The investigator may authorize other medical personnel he/she trusts to act as appropriate sub-investigators to assist in the conduct of the clinical study according to the requirements of the clinical protocol. All sub-investigators will conduct the study under the supervision of the investigator. The investigator will provide them with a copy of the clinical trial protocol and all necessary information.

12.2 Sponsor Responsibilities

The sponsor of this clinical trial shall be responsible for the national regulatory authorities, and take all reasonable measures to ensure the moral ethics and compliance of the clinical trial as well as the authenticity and integrity of the case report form data records. Therefore, the main responsibility of the monitor is to assist the investigator and the sponsor to maintain a high level of moral, scientific, technical and regulatory quality in all aspects of the clinical trial.

During the course of the clinical trial, the monitor will periodically contact the trial site by visit, mail or telephone to review the progress of the study, the compliance of the investigator and patients with the clinical trial protocol and various urgent problems. During these monitoring visits, the monitor will review with the investigator the following details: patient informed consent forms, patient enrollment and follow-up, patient compliance with the study drug regimen, study drug assignment, study drug accountability, use of concomitant medications and other therapies, and data quality, SAE recording and reporting, and data quality.

13 DATA MANAGEMENT

13.1 Requirements for original documents

In accordance with ICH GCP guidelines, monitors must verify the eCRF against source documents unless it is pre-specified that source information is to be recorded directly on the electronic case report form (eCRF). The authorized person of the sponsor, the Ethics Committee and regulatory authorities can directly view the original documents of the data recorded in the eCRF (such as the patient's medical file, appointment book, original laboratory test results, etc.). These persons, subject to the principle of occupational confidentiality, must keep the identity or medical information of all subjects confidential.

13.2 Case Report Forms and Precautions

The EDC system was used in this study to process clinical trial data. The case report forms (CRFs) for this study will be in electronic data format, depending on the method of data collection.

Each subject had a separate, complete eCRF form. All completed original eCRF forms are the sole property of Shanghai Miracogen Inc. Unless authorized by Shanghai Miracogen Inc. or relevant government authority, any third party cannot obtain and use the data in eCRF.

It is the responsibility of the investigator to complete all trial data required by this protocol on the eCRF forms. The investigator is also obliged to ensure that the data in the eCRF form are consistent with those in the original medical records. The eCRF is a controlled document. The investigator must sign the eCRF and ensure the authenticity, accuracy and integrity of the data. If modification is required, the modification record information will be automatically recorded in the EDC system.

The clinical research associate (CRA) should ensure the authenticity, accuracy and completeness of the data by verifying the original medical records, checking the completed eCRF and other study-related information during each monitoring visit.

13.3 Application of Computer System

A computerized system will be used to generate, modify, maintain, archive, and restore all data collected in the clinical study.

13.4 Data collection

This study will collect clinical study data using the EDC system. Case form forms (CRFs) for this study will be in electronic data format.

13.5 Data Management and Quality Control

The data administrator shall, together with the principal investigator, propose the contents of data range check and logic check according to the range and interrelation of each indicator value in the case report form. Compile the corresponding computer program, control the input of wrong data before input, find out the cause of error and make correction. All the error contents and modification results shall be recorded and properly preserved.

Data verification will be divided into manual verification and system verification. Manual verification is medical verification, which checks the consistency and logic of data from the perspective of medical specialty. System verification is to verify the database by writing SAS program and automatically sort out the data that does not meet the requirements of clinical study by running the system program. For any question in the eCRF, DM will generate a question in EDC. The investigator should answer the question in EDC system as soon as possible and possibly modify the data. DM will confirm whether to close the question based on the investigator's answer and the updated data, and can raise the question again if necessary, until all the questions are resolved. All modifications and updates will be documented and archived.

CDISC standards were implemented for data management in this study.

13.6 Data Retention

In the face of inspections and audits by government regulatory authorities and Shanghai Miracogen Inc., the investigator agrees to retain all original documents, photocopies, meeting minutes and physical objects related to the subjects in this study. It is recommended that the investigator retain the study file for at least 15 years after the trial is completed or halfway stopped. If the Investigator is no longer able to retain the study documentation, the Sponsor must be notified and the relevant documentation transferred to a third party acceptable to both parties. Within 15 years after the end or discontinuation of the clinical trial, the investigator must obtain a written letter of authorization from the sponsor before disposing or destroying the study data and archives.

13.7 Modification to Test Protocol

The investigator should not deviate from the current clinical trial protocol without sponsor approval and prior approval/favorable opinion or filing of the amendments by the Clinical Institutional Review Board and Ethics Committee, unless the changes eliminate an immediate hazard to study subjects or the changes involve only logistical or administrative aspects of the trial. Any amendment agreed should be documented and the written amendment should be signed by both the investigator and the sponsor, and archived together with the clinical trial protocol.

In some cases, it may be necessary to modify the informed consent form at the same time. The investigator must approve/acknowledge the written ICF amendment by IRB and EC before implementing the amendment.

13.8 Principles for publication and sharing of trial results

The investigator has the right to use the results obtained from the clinical trial for teaching and communication in meetings and publications. In order to ensure the accuracy and scientificity of information, while protecting the independence and responsibility of investigators and protecting the confidentiality of information, only verified and valid materials can be used. For this reason, any publication or communication, or draft publication or communication by the Investigator shall be preceded by communication and discussion among all parties. Therefore, the investigator undertakes and ensures that all sub-investigators undertake not to make any publication, communication or release of the results of the study without the written consent of the sponsor.

The investigator must provide the sponsor with a copy of the manuscript for review at least 45 days prior to journal submission or 20 days prior to abstract submission. Publication must be postponed for no more than 90 days before a written response is received from the sponsor, which is understood to mean that the sponsor cannot refuse publication without proper reason. The investigator agrees to the modifications requested by the sponsor, which should not jeopardize the accuracy or scientificity of the publication.

In a multi-center study, the investigator agrees not to publish the relevant results of his/her study site before the overall study results are published. If no results are published within 12 months after the termination of the clinical trial in all the study sites, the investigator has the right to independently publish the results of the clinical trial in this study site, and the review procedure is described as before. If the clinical trial is managed by a steering committee, the latter may develop detailed provisions for publication.

The investigator should not use the name of the sponsor and/or its employees in advertising or promotional materials or publications without the written consent of the sponsor. The Sponsor shall not use the names of the Investigator and/or his/her colleagues in advertising or promotional materials or publications without the written consent of the Subject or Investigator.

The sponsor has the right to publish the study results at any time.

14 STATISTICAL ANALYSIS

14.1 Sample Size

The number of patients with advanced solid tumors in Phase Ia needs to be determined based on the results of patient safety evaluation in the actual dose escalation trial. The expected number of patients is approximately 26.

A total of 36 patients (12 in each category) with advanced colorectal cancer, head and neck squamous cell carcinoma, and nasopharyngeal carcinoma were evaluable for the phase Ib trial. Since this study is an exploratory Phase I clinical trial, the number of patients participating (i.e., sample size) is not based on formal statistical calculations.

14.2 Statistical Analysis Plan

Statistical analysis was performed using SAS Enterprise version 5.1 (or higher) software. The study results will be summarized and analyzed by descriptive statistical methods for safety indicators and efficacy indicators.

Kinetic parameters include but are not limited to C_{max}, T_{max}, $t_{1/2}$, MRT, V_d, CL, AUC_{0-t} and AUC_{0-inf}, etc.; kinetic analysis is performed according to non-compartmental model; concentration and kinetic parameter pharmacokinetic analysis software is used for evaluation.

The analytical parameters of immunogenicity include ADA positive rate and antibody.

The detailed statistical analysis strategy and steps will be described in a separate Statistical Analysis Plan (SAP). After the start of the trial, for the modification of primary study objectives and important secondary study objectives, or changes in corresponding statistical analysis methods, the protocol and statistical analysis plan need to be amended and the final version should be obtained before database lock. After database lock, additional exploratory analyses not included in the protocol or statistical analysis plan will be described in detail in the clinical trial report (CSR).

14.3 Data Analysis Set

Safety Set (SS): includes all subjects who received at least one treatment. Safety analysis set is used for safety analysis and primary analysis of efficacy indicators.

Evaluable Analysis Set: Phase Ia includes subjects who have at least one post-treatment tumor assessment; Phase Ib includes subjects who have not achieved CR and PR response and have at least one evaluable baseline post-treatment tumor assessment, and subjects who have achieved CR and PR response include at least two post-treatment tumor assessments (and are at least 4 weeks apart). The evaluable analysis set was used for secondary analyses of efficacy measures.

PK Analysis Set (PKS): includes subjects who received at least one treatment and had at least one valid post-treatment PK concentration measurement. The PK analysis set was used for the analysis of PK parameters.

14.4 Statistical Analysis Methods

14.4.1 Overall Analytical Method

The results of this study will be analyzed and summarized using descriptive statistical methods. Measurement data will be subject to descriptive statistical analysis using mean, standard deviation, median, minimum and maximum. PK parameters will also be listed with geometric mean and coefficient of variation. Enumeration data or grade data will be expressed with frequency and frequency.

14.4.2 Baseline Analysis Methods

Items collected during the baseline period included:

- Subject characteristics at baseline: demographics (age, gender, ethnicity), height, weight, disease-related symptoms, ECOG score, laboratory values, gene expression or gene mutation;
- Tumor characteristics at inclusion: duration of disease, histopathological type, tumor stage, resection of primary tumor and site of tumor metastasis;
- History of prior anticancer therapy: surgery, chemotherapy, radiotherapy, or other anticancer therapy.

For continuous variables of age, height and weight, the statistics of mean, standard deviation, median, maximum and minimum of each variable are given; for categorical variables of gender and ethnicity, the frequency of each type and the corresponding percentage are given. At the same time, the medical history, other current diseases and medication were statistically described.

14.4.3 Analysis method of primary outcome measures

14.4.3.1 Primary Outcome Measures in Part Ia

To observe the frequency of dose limiting toxicity (DLT) in subjects receiving different doses of intravenous drip of MRG003 within the first cycle. Doses at which the MTD or RP2D is inferred (see Section 6.3.4).

14.4.3.2 Primary Outcome Measures in Part Ib

According to RECIST v1.1, evaluate the treatment indicators related to tumor measurement: objective response rate (ORR = 100% x (CR + PR)/total number of subjects in analysis set), and calculate the corresponding 95% CI.

14.4.4 Analysis method of secondary outcome measures

14.4.4.1 <u>Secondary Outcome Measures in Phase Ia</u>

- PK parameters of all subjects in the three components of MRG003 were analyzed by non-compartmental method during the dosing period, but not limited to C_{max}, T_{max}, $t_{1/2}$, MRT, V_d, CL, $AUC_{0\text{-}t}$ and $AUC_{0\text{-}inf}$.
- Drug safety and tolerability were evaluated. Descriptive statistical methods were used to analyze and summarize the type, severity, occurrence time, frequency and correlation of adverse events with the study drug.
- Anti-therapeutic drug antibody (ADA) analysis: Descriptive statistical analysis was performed using the percentage of ADA positive, and the range of each ADA positive subject was listed.
- After intravenous drip of MRG003 at different doses every two treatment cycles, the best efficacy response, complete response (CR), partial response (PR), stable disease (SD), progressive disease (PD) and non-evaluable (NE), was assessed in subjects throughout the trial. The number and proportion of subjects with each response will be summarized and described. Confirmation of two response evaluations is not required for best response in Phase Ia. If a subject has stable disease (SD) as their best response, SD must be evaluated at least 5 weeks after the first dose. The objective response rate (ORR = 100% x (CR + PR)/total number of subjects in the analysis set) and its corresponding 95% CI will be calculated.

14.4.4.2 <u>Secondary Outcome Measures in Phase Ib</u>

- Drug safety and tolerability will be assessed. Descriptive statistical methods will be used to analyze and summarize the types and severity of adverse events and their correlation with the study drug.
- At MTD/RP2D, within the treatment cycle of intravenous drip of MRG003, determine the concentrations of the three drug components in all subjects, and calculate the drug PK values, not limited to C_{max}, T_{max}, $t_{1/2}$, MRT, V_d, CL, $AUC_{0\text{-}t}$ and $AUC_{0\text{-}inf}$; perform kinetic analysis according to non-compartment model; evaluate the concentration and pharmacokinetic parameter pharmacokinetic analysis software.
- Anti-therapeutic drug antibody (ADA) analysis: Descriptive statistical analysis was performed using the percentage of ADA positive, and the range of each ADA positive subject was listed.
- To assess the best response of subjects throughout the trial according to RECIST v1.1 criteria: complete response (CR), partial response (PR), stable disease (SD), progressive disease (PD) and non-evaluable (NE). The number and proportion of subjects in each efficacy endpoint will be summarized and described. The best response in Phase Ib requires confirmation by two assessments.

- To assess tumor efficacy measures in relation to time: progression-free survival (PFS), overall survival (OS), and duration of response (DOR). The product limit method was used to calculate the estimated survival time and 95% confidence interval, and the survival time and its confidence interval in the first and third quartiles (Q1, Q3). Survival curves were plotted with the Kaplan-Meier method.

14.4.5 Safety Analysis Methods

For safety analysis, descriptive statistics will be used to summarize incidence of adverse events (AEs) and calculate normal/abnormal changes of laboratory test results before and after treatment. The listing of various AEs and the listing of clinically significant abnormalities in laboratory parameters after treatment will be detailed.

Safety indicators will include adverse events, vital signs (blood pressure, pulse and body temperature) and laboratory data. Safety analysis will be based on Safety Analysis Set (SS).

The analysis of adverse events in the safety analysis only analyzed treatment-emergent adverse events (TEAEs). For adverse events (TEAEs) during the treatment period, all AEs occurred within 21 days after the last dose, and adverse events and serious adverse events were collected. Adverse events will be summarized, and the number and percentage of patients with adverse events and the number of events will be calculated, classified by MedDRA preferred term, and severity will be determined based on CTCAE v.4.03 grading. The list of adverse events will be listed according to the occurrence time of adverse events of subjects, the treatment period where they are located, and the CTCAE grade of adverse events.

Hematological toxicity was assessed with laboratory parameters. Neutropenia, thrombocytopenia, and anemia were graded by CTCAE and calculated according to the National Cancer Institute (NCI) Common Terminology Criteria. Qualitative and quantitative results of hematological toxicity will also be summarized.

The blood biochemical indicators of the subjects were assessed according to the blood biochemical reference values of the study hospital. Blood biochemical indicators were analyzed and summarized according to CTCAE grade.

15 QUALITY ASSESSMENT AND MANAGEMENT

The principal investigator and the clinical study institution allow the CRO, sponsor, ethics committee/clinical institution review committee and authorized representatives of government regulatory authorities to monitor, audit and inspect the clinical trial. These authorized representatives may also review the original clinical study documents and data directly.

15.1 Trial Monitoring

The monitor should contact and visit the investigator regularly to evaluate the progress of the trial and the compliance with GCP, regulations and trial protocol, and collect and confirm the authenticity, accuracy and comprehensiveness of the data recorded in the eCRF. The investigator and the personnel involved in the trial should cooperate with the monitor to ensure that the problems that may be found are resolved.

15.2 Trial Audits

The main purpose of the audit is to confirm that the rights and safety of patients are protected, and that all data related to the evaluation of investigational drugs are operated and reported in accordance with GCP and relevant administrative regulations. Covance Pharmaceutical R & D (Beijing) Co., Ltd. is responsible for auditing the implementation of clinical trials to assess compliance with the Good Clinical Practice (GCP).

15.3 Study inspection

Government regulatory authorities may also initiate an inspection of a clinical trial during or after the trial is completed, and if the investigator receives such notice, he/she should immediately notify Shanghai Miracogen Inc. to cooperate with the relevant work.

16 ETHICS RELATED

This clinical trial will be conducted in accordance with the Declaration of Helsinki (2000 Edition) and the relevant Good Clinical Practice and regulations in China. The study protocol can be implemented only after approved by the Ethics Committee of Sun Yat-sen University Cancer Center before the study is initiated. If this trial involves multiple clinical study sites, the Ethics Committee of each clinical study site shall approve the clinical trial protocol to be implemented in that site. The investigator and sponsor were not able to dispense study drug and the investigator was not able to initiate the study until written and dated approval/consent was obtained. During the clinical trial, any amendment to the trial protocol should be approved or filed by the Ethics Committee before implementation. Any event that is likely to affect the safety of patients or the continuation of the clinical trial, especially any change in safety, should also be reported to the Ethics Committee. Any update to the Investigator's Brochure should be submitted to the Ethics Committee. The clinical investigator must inform the subject that participation in the clinical trial is voluntary, that the subject has the right to withdraw from the trial at any stage of the trial without discrimination or retaliation, that his/her medical treatment and rights and interests are not affected, and that he/she can continue to receive other treatment or treatment. The subjects must be informed that the personal data of participating in the trial and during the trial are confidential. The subject should be informed of the nature of the clinical trial, the purpose of the trial, the expected possible benefits and possible risks and inconveniences, and of other treatment options available to the subject and the rights and obligations of the subject in accordance with the Declaration of Helsinki, so that the subject can have sufficient time to consider whether he/she is willing to participate in the trial,

16.1 Signature of Informed Consent Form

All patients had to sign a written Patient Informed Consent Form (ICF) before agreeing to participate in the trial. In addition, in Phase Ib of this study, patients are required to sign an additional Informed Consent Form for Biomarker Screening for EGFR testing prior to screening. In this study, the informed consent form signed by the patient should contain two parts: the informed consent of the patient to participate in this study; and the informed consent of the patient to the relevant genetic testing. The informed consent document shall be submitted to the Ethics Committee for review and approval together with the clinical trial protocol, and shall be notified of the approval before the start of the study.

The investigator should inform the subjects of the objective, process, possible risks and benefits of this trial and other details of the clinical trial in both oral and written forms. The subject should read the "Informed Consent Form" carefully. At the same time, the relevant questions were verbally explained by the researchers. The subject must be given sufficient time to inquire about trial details. If the subject is limited by his/her ability to fully understand the trial-related matters, the subject's legal representative shall be present. The legal representative shall participate in the whole process of informed consent together

with the subject. According to the subject's comprehension, the subject shall be informed of the trial to the greatest extent.

If the subject or his/her legal representative voluntarily participates in this trial, he/she should sign and date the Patient Informed Consent Form. The investigator who conducted the informed consent process must also sign and date the ICF.

If the subject and his/her legal representative are illiterate, an impartial witness should be present during the entire informed consent discussion. After the subject has orally agreed to participate in the trial and his or her representative has signed and dated the informed consent form, the impartial witness signs and dates the form to attest that all trial contents have been explained to the subject or the subject's legal representative.

The subject was informed that he or she could not participate in the trial and could withdraw unconditionally at any stage of the trial, even if he or she had participated. He or she should also be informed that if he or she refuses to participate in the trial or withdraws from the trial, his or her rights and interests will not be affected.

The signed ICF will be kept in duplicate for each subject and site.

If important new information involving the investigational drug is found, the ICF should be revised. After reporting to the Ethics Committee for approval, the consent of the subject or his/her legal representative should be obtained again.

16.2 Protect patient privacy

Unless otherwise specified by law, the subject's name shall not appear in all study materials, publications or other relevant statements of the sponsor. In the Sponsor's files, subjects will be identified only by initials and a code number. When the data involved in the study are transmitted in different units or regions, the personal data of the subjects shall be regarded as the highest confidentiality.

17 INTELLECTUAL PROPERTY

The Sponsor has its own independent intellectual property rights for all information, documents or study drugs provided by the Sponsor or its designee. The investigator cannot refer to any information of the drug in the therapeutic drug or other intellectual property rights, nor apply for the property rights or other intellectual property rights. All results, data and inventions in any form directly or indirectly obtained from clinical trials are independent property rights of Shanghai Miracogen Inc. All results may be used or applied at the discretion of the sponsor without restriction on title (scope, field, shelf life). The Sponsor is not obligated to patent, develop, sell or otherwise use the results of the clinical trial. If applicable, the investigator and/or sub-investigator should provide the sponsor with all the assistance necessary to obtain and maintain any patents, including the signature of legal documents, at the sponsor's expense.

18 REFERENCES

1. Carlos L. Arteaga. Epidermal Growth Factor Receptor Dependence in Human Tumors: More Than Just Expression? [J]. The Oncologist, 2002, 7 (4): 31-39.
2. Stephanie K.A. Blick, Lesley J. Scott. Cetuximab. A Review of its Use in Squamous Cell Carcinoma of the Head and Neck and Metastatic Colorectal Cancer [J]. Drugs, 2007, 67 (17): 2585-2607.
3. Roy S. Herbst, Dong M. Shin. Monoclonal Antibodies to Target Epidermal Growth Factor Receptor – Positive Tumors [J]. Cancer, 2002, 94 (5): 1593-1611.
4. Kosaka et al. Mechanisms of Resistance to EGFR TKIs and Development of a New Generation of Drugs in Non-Small-Cell Lung Cancer. Journal of Biomedicine and Biotechnology, 2011:1-7 pages.
5. Kedan Lin, Jay Tibbitts, and Ben-Quan Shen. Pharmacokinetics and ADME Characterizations. Methods in Molecular Biology, 2013, 1045:117-131,
6. Haleh Saber, John K. Leighton. An FDA oncology analysis of antibody-drug conjugates. Regulatory Toxicology and Pharmacology, 2015 (71): 444-452.
7. Ben-Quan Shen, Keyang Xu, Luna Liu, et al. Conjugation site modulates the in vivo stability and therapeutic activity of antibody-drug conjugates. Nature Biotechnology, 2012, 30 (2): 184-191.
8. Xu Ruihua, Luyan. Progress in Targeted Therapy for Colorectal Cancer [J]. China Pharmacy, 2010, 21 (14): 1254-1257.
9. Yang Yaqiong, Li Zonghai. Research Progress of Tumor Molecular Targeting Drugs Targeting EGFR [J]. Chinese Journal of Bioengineering, 2012, 32 (5): 91-96.
10. Wang Li, Yu Yinghao. Current status of k-ras gene detection and targeted therapy for colorectal cancer [J]. World Chinese Journal of Gastroenterology, 2011, 19 (1): 62-67.
11. Wang Caixia, Xie Junxia, Mi Yi, Zhao Qian. Progress in the Study of Alfa, a New Drug for the Treatment of Cell Lung Cancer [J]. Journal of Shenyang Pharmaceutical University, 2015, 32 (3): 245-248.

19 APPENDICES

19.1 Appendix I: Performance Status Scoring Criteria (ECOG)

Level	Statement of performance status
**0**	Fully active, able to carry on all pre-disease performance without restriction;
**1**	Restricted in physically strenuous activity but ambulatory and able to carry out work of a light or sedentary nature, e.g., light house work, office work;
**2**	Ambulatory and capable of all self-care but unable to carry out any work activities. Up and about more than 50% of waking hours;
**3**	Capable of only limited self-care, confined to bed or chair more than 50% of waking hours;
**4**	Bedridden, unable to carry on any self-care;
**5**	Death

Quoted from: Am. J. Clin. Oncol. (CCT) 1982; 5:649-655

19.2 Appendix II: New Response Evaluation Criteria in Solid Tumors: Revised RECIST Guidelines (Version 1.1)

Measurable lesions:

Tumor lesions: must be accurately measured in at least one dimension (longest diameter in the plane of measurement is to be recorded) with a minimum size of:

- 10 mm by CT scan or MRI (CT scan slice thickness no greater than 5 mm)
- Or
- 10 mm caliper measurement by clinical exam (lesions which cannot be accurately measured with calipers should be recorded as non-measurable)
- Or
- Chest X-ray 20 mm Malignant lymph nodes: Pathologically enlarged and measurable lymph nodes must be ≥ 15 mm in short axis when assessed by CT scan (CT scan slice thickness recommended to be no greater than 5 mm). At baseline and follow-up, only the short axis will be measured and followed.

Non-measurable lesions:

All other lesions, including small lesions (longest diameter < 10 mm or pathological lymph nodes with ≥ 10 to < 15 mm short axis) and truly non-measurable lesions. Lesions

considered truly non-measurable include: leptomeningeal disease, ascites, pleural or pericardial effusion, inflammatory breast disease, lymphangitic involvement of skin/lung, abdominal enlargement or abdominal organomegaly identified by physical examination that is not measurable by repeat imaging techniques.

Target Lesions:

When more than one measurable lesion is present at baseline, all lesions up to a maximum of five lesions total (and a maximum of two lesions per organ) representative of all involved organs should be identified as target lesions.

Target lesions must be selected on the basis of their size (longest diameter), be representative of all involved organs, and be measured with good reproducibility.

A sum of the diameters (longest for non-nodal lesions, short axis for nodal lesions) for all target lesions will be calculated and reported as the baseline sum diameters. If lymph node diameter is included, only the short axis is included.

Non-target lesions:

All remaining lesions (or sites of disease) including pathological lymph nodes should be identified as non-target lesions and should not be measured but should be recorded at baseline assessment and recorded as "present", "absent" or in rare cases "unequivocal progression".

For patients with measurable target lesions, overall response will be assessed at the time of tumor measurement as detailed in the table below.

Overall Response Assessment in Patients with Measurable Disease at Baseline

Target Lesions	Non-target lesions	New lesions	Overall response
CR	CR	None	CR
CR	Non-CR/Non-PD	None	PR
CR	NE	None	PR
PR	Non-PD or NE	None	PR
SD	Non-PD or NE	None	SD
Not all lesions can be evaluated	Non-PD	None	NE
PD	Any condition	Yes or No	PD
Any condition	PD	Yes or No	PD
Any condition	Any condition	Yes	PD

Abbreviations: CR = complete response; PR = partial response; SD = stable disease; PD = progressive disease; NE = not evaluable

For patients without measurable disease (i.e., non-target disease), overall response will also be assessed at the time of tumor measurement as detailed in the table below.

Overall Response Assessment in Patients without Measurable Disease at Baseline

Non-target lesions	New lesions	Overall response
CR	None	CR
Non-CR/Non-PD	None	Non-CR/Non-PD
Not all lesions can be evaluated	None	NE
Unequivocal PD	Yes or No	PD
Any condition	Yes	PD

Abbreviations: CR = complete response; PD = progressive disease; NE = not evaluable

**Target Lesion Assessment**	
Complete response (CR)	All target lesions disappear and all pathological lymph nodes (including target and non-target nodules) must have reduction in short axis to < 10 mm.
Partial response (PR)	At least a 30% decrease in the sum of diameters of target lesions, taking as reference the baseline sum of diameters of target lesions.
Stable disease (SD)	Neither sufficient shrinkage to qualify for PR nor sufficient increase to qualify for PD, taking as reference the smallest sum diameters while on study.
Progressive Disease (PD)	Throughout the study, at least a 20% relative increase in the sum of diameters of target lesions, taking as reference the smallest sum on study (this includes the baseline sum if that is the smallest on study); in addition to the relative increase of 20%, the sum must also demonstrate an absolute increase of at least 5 mm (the appearance of one or more new lesions is also considered progression).
**Assessment of Non-Target Lesions**	
Complete response (CR)	Disappearance of all non-target lesions and normalization of tumor markers. All lymph nodes must be non-pathological in size (< 10 mm short axis).
Non-CR/Non-PD	Presence of one or more non-target lesions and/or persistence of tumor marker level above the normal level.
Progressive Disease (PD)	Unequivocal progression of existing non-target lesions. (Note: The appearance of one or more new lesions is also considered progression).

19.3 Appendix III: Cancer Drug Standard Terminology Adverse Event Evaluation System (CTCAE v4.03)

Common Terminology Criteria for Adverse Events (CTCAE v4.03: June 14, 2010) published by the National Cancer Institute.
