# Supplementary material for: Evaluation of Safety of Treatment With Anti–Epidermal Growth Factor Receptor Antibody Drug Conjugate MRG003 in Patients With Advanced Solid Tumors: A Phase 1 Nonrandomized Clinical Trial
Source: JAMA Oncol. 2022 May 5;8(7):1042–6. doi: 10.1001/jamaoncol.2022.0503 (PMC9073657; doi:10.1001/jamaoncol.2022.0503)
Supplement: Supplement 2. — eTable 1. Pharmacokinetics for phase Ia dose-escalation and phase Ib dose-expansion eTable 2. Treatment-related AEs (TRAEs) ≥ Grade 3 of all patients eTable 3. The relationships between EGFR status, BOR, PFS, and OS eFigure 1. Pharmacokinetics of MRG003 following the first administration eFigure 2. Progression-free survival for phases Ib dose-expansion [file jamaoncol-e220503-s002.pdf]

Supplemental Online Content

Qiu M-Z, Zhang Y, Guo Y, et al. Evaluation of safety of treatment with anti–epidermal growth factor receptor antibody drug conjugate MRG003 in patients with advanced solid tumors: a phase 1 nonrandomized clinical trial. *JAMA Oncology*. Published online May 5, 2022. doi:10.1001/jamaoncol.2022.0503

eTable 1. Pharmacokinetics for phase Ia dose-escalation and phase Ib dose-expansion

eTable 2. Treatment-related AEs (TRAEs) ≥ Grade 3 of all patients

eTable 3. The relationships between EGFR status, BOR, PFS, and OS

eFigure 1. Pharmacokinetics of MRG003 following the first administration

eFigure 2. Progression-free survival for phases Ib dose-expansion

This supplemental material has been provided by the authors to give readers additional information about their work.

© 2022 Qiu M-Z et al. *JAMA Oncology.*

eTable 1. Pharmacokinetics for phase Ia dose-escalation and phase Ib dose-expansion

		Ia															Ib					
		0.1 mg/kg		0.3 mg/kg		0.6 mg/kg		1.0 mg/kg		1.5 mg/kg		2.0 mg/kg		2.5 mg/kg			SCCHN		NPC		mCRC	
		n	Mean	n	Mean	n	Mean (CV%)	n	Mean (CV%)	n	Mean (CV%)	n	Mean (CV%)	n	Mean (CV%)		n	Mean (CV%)	n	Mean (CV%)	n	Mean (CV%)
Cmax (ng/mL)	MMAE	1	0.0887	1	0.467	3	2.18	3	1.93	3	2.54	4	5.36	7	7.39		13	8.34	14	7.14	12	5.52
	MRG003	1	1420	1	4190	3	9390	3	30200	3	35800	4	51500	7	64300		13	81200	14	72900	12	74300
	Tab	1	1190	1	3680	3	9220	3	21600	3	26900	4	40300	7	57900		13	61200	14	54400	12	55400

© 2022 Qiu M-Z et al. *JAMA Oncology.*

Tmax (h)	MMAE	1	24.5	1	24.5	3	49.00	3	25.0	3	49.00	4	73.01	7	98.00		13	72.15	14	72.18	12	72.71
	MRG003	1	0.5	1	0.52	3	1.00	3	1.00	3	1.03	4	1.03	7	2.03		13	2.07	14	2.02	12	2.03
	Tab	1	0.5	1	2.53	3	1.00	3	1.00	3	1.03	4	2.02	7	2.03		13	2.12	14	2.02	12	2.03
AUC_{0-t} (h.ng/mL)	MMAE	1	4.37	1	59.5	3	330	2	502.5	3	490	2	942	3	1190		11	1950	9	1370	8	1040
	MRG003	1	7200	1	85200	3	192000	2	853000	3	957000	3	1920000	5	2930000		12	3190000	10	3120000	9	3280000
	Tab	1	10600	1	103000	3	291000	2	1260000	3	1250000	3	2760000	5	3690000		12	4030000	10	3650000	9	4320000

© 2022 Qiu M-Z et al. *JAMA Oncology.*

AUC_{0-inf} (h.ng/mL)	MMAE	0	NC	1	60.7	3	335	3	375	3	494	3	834	4	1430		11	1860	9	1220	9	1140
	MRG003	1	8270	1	86800	3	195000	3	855000	3	960000	4	1890000	7	2880000		13	3280000	14	3100000	12	3270000
	Tab	1	12300	1	104000	3	298000	3	1140000	3	1290000	4	2750000	7	3690000		13	4100000	13	3700000	11	4310000
$t_{1/2}$ (h)	MMAE	0	NC	1	55.2	3	59.0	3	60.4	3	65.2	4	88.8	4	60.1		11	59.2	9	61.6	9	74.7
	MRG003	1	9.1	1	16.7	3	14.9	3	27.3	3	29.0	4	35.3	7	37.2		13	33.9	14	36.4	12	35.9
	Tab	1	9.5	1	12.3	3	16.5	3	21.8	3	29.0	4	29.1	7	40.1		13	35.9	14	42.6	12	40.5

© 2022 Qiu M-Z et al. *JAMA Oncology.*

V_D (L)	MRG003	1	8.74	1	4.75	3	3.55	3	2.75	3	3.65	4	2.83	7	2.86		13	2.39	14	2.53	12	2.46
	Tab	1	6.14	1	2.9	3	2.57	3	1.65	3	2.72	4	1.61	7	2.41		13	2.03	14	2.44	12	2.08
CL (L/h)	MRG003	1	0.665	1	0.197	3	0.165	3	0.0698	3	0.0872	4	0.0556	7	0.0533		13	0.0490	14	0.0483	12	0.0475
	Tab	1	0.448	1	0.164	3	0.108	3	0.0524	3	0.0649	4	0.0383	7	0.0416		13	0.0392	14	0.0397	12	0.0355

T_{max} is shown as the median.

C_{max}: maximum serum concentration; T_{max}: time to reach maximum serum concentration; AUC: area under the curve; t1/2: elimination half time; V_D: distribution volume; CL: clearance; MMAE: monomethyl auristatin E; Tab: total antibody.

© 2022 Qiu M-Z et al. *JAMA Oncology.*

eTable 2. Treatment-related AEs (TRAEs) ≥ Grade 3 of all patients

TRAE	N (%)
Hyponatremia	5 (8%)
Decreased white blood cell count	4 (7%)
Decreased neutrophil count	3 (5%)
Increased aspartate aminotransferase	2 (3%)
Febrile neutropenia	2 (3%)
Decreased platelet count	2 (3%)
Hypokalemia	1 (2%)
Anemia	1 (2%)
Dyspnea	1 (2%)
Respiratory failure	1 (2%)
Lung infection	1 (2%)
Rash	1 (2%)
Pruritus	1 (2%)
Maculo-papular rash	1 (2%)
Increased alanine aminotransferase	1 (2%)

© 2022 Qiu M-Z et al. *JAMA Oncology.*

Decreased weight	1 (2%)
Increased blood pressure	1 (2%)
Increased bilirubin	1 (2%)
Multiple organ dysfunction syndrome	1 (2%)
Oral mucositis	1 (2%)
Pain in extremity	1 (2%)
Hypoaesthesia	1 (2%)
Peripheral neuropathy	1 (2%)
Headache	1 (2%)
Metabolic acidosis	1 (2%)
Hypophosphatemia	1 (2%)
Hypocalcemia	1 (2%)
Hyperglycaemia	1 (2%)
Interstitial lung disease	1 (2%)
Hypotension	1 (2%)

© 2022 Qiu M-Z et al. *JAMA Oncology.*

eTable 3. The relationships between EGFR status, BOR, PFS, and OS

EGFR level	No.	BOR					Phase Ib	
		CR	PR	SD	PD		PFS(month)	OS(month)
1+	4	0	1	1	2		2.8	10.9
2+	10	0	0	5	5		2.0	9.5
3+	6	0	2	3	1		2.7	11.8
4+	14	0	6	6	2		4.1	Not reached

EGFR: epidermal growth factor receptor; BOR: best overall response; PFS: progression-free survival; OS: overall survival; CR: complete response; PR: partial response; SD: stable disease; PD: progression disease

© 2022 Qiu M-Z et al. *JAMA Oncology.*

eFigure 1. Pharmacokinetics of MRG003 following the first administration

A

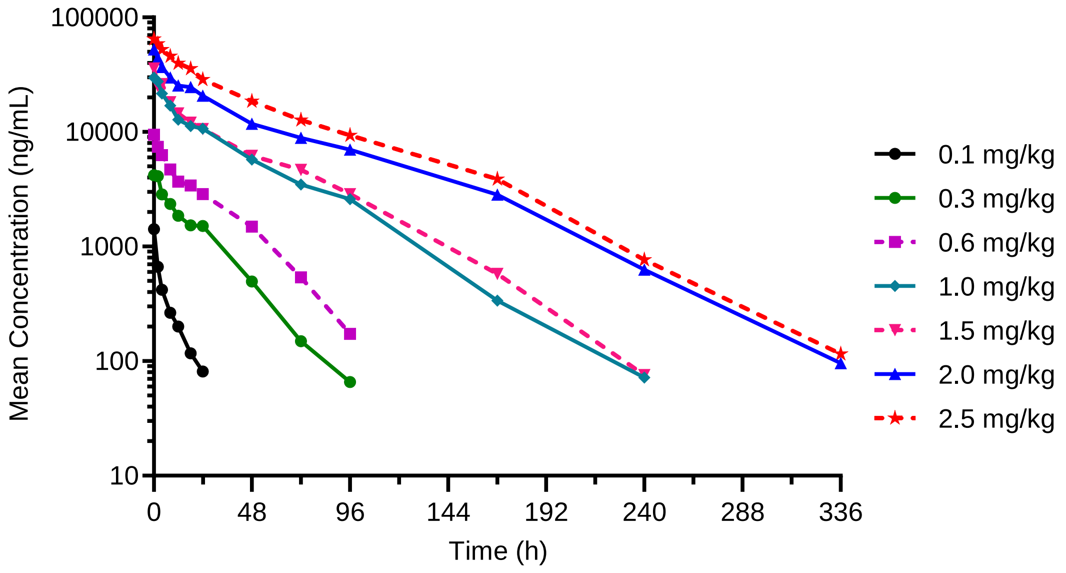

B

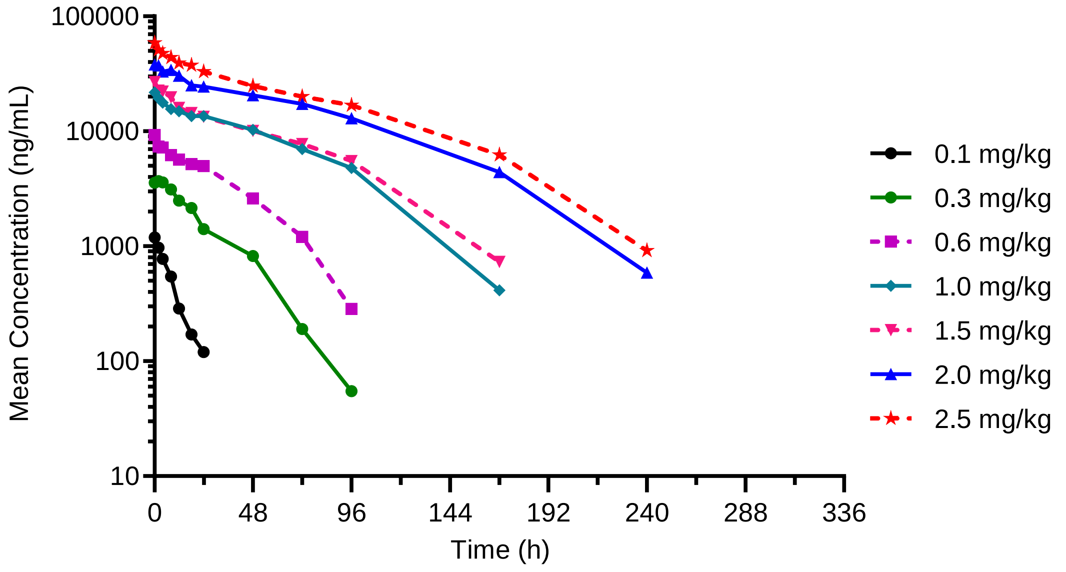

© 2022 Qiu M-Z et al. *JAMA Oncology.*

C

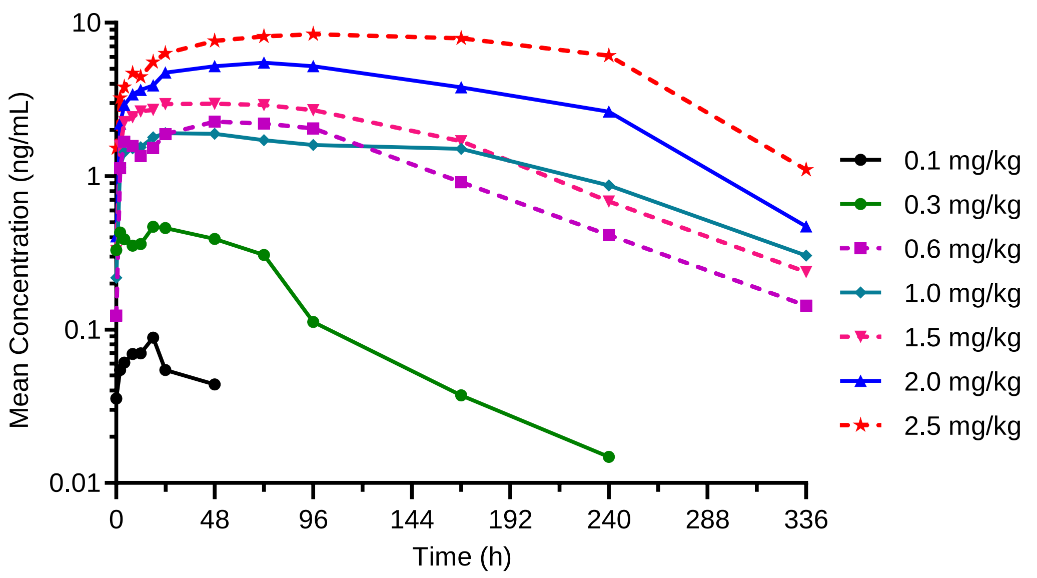

D

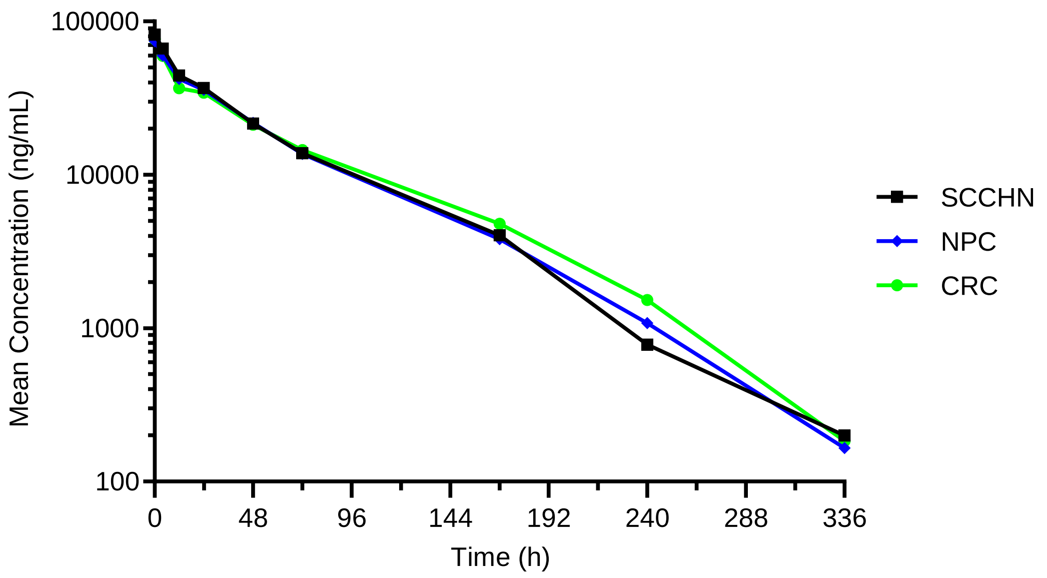

Supplementary figure 1 A-C show serum concentrations of MRG003, total antibody (Tab) and monomethyl auristatin E (MMAE) over 14 days (logarithmic graph). 1 D show serum concentrations of MRG003 over 14 days in patients with squamous cell carcinoma of head neck cancer (SCCHN), nasopharyngeal cancer (NPC) and colorectal cancer (mCRC) (logarithmic graph).

© 2022 Qiu M-Z et al. *JAMA Oncology.*

eFigure 2. Progression-free survival for phase Ib dose-expansion

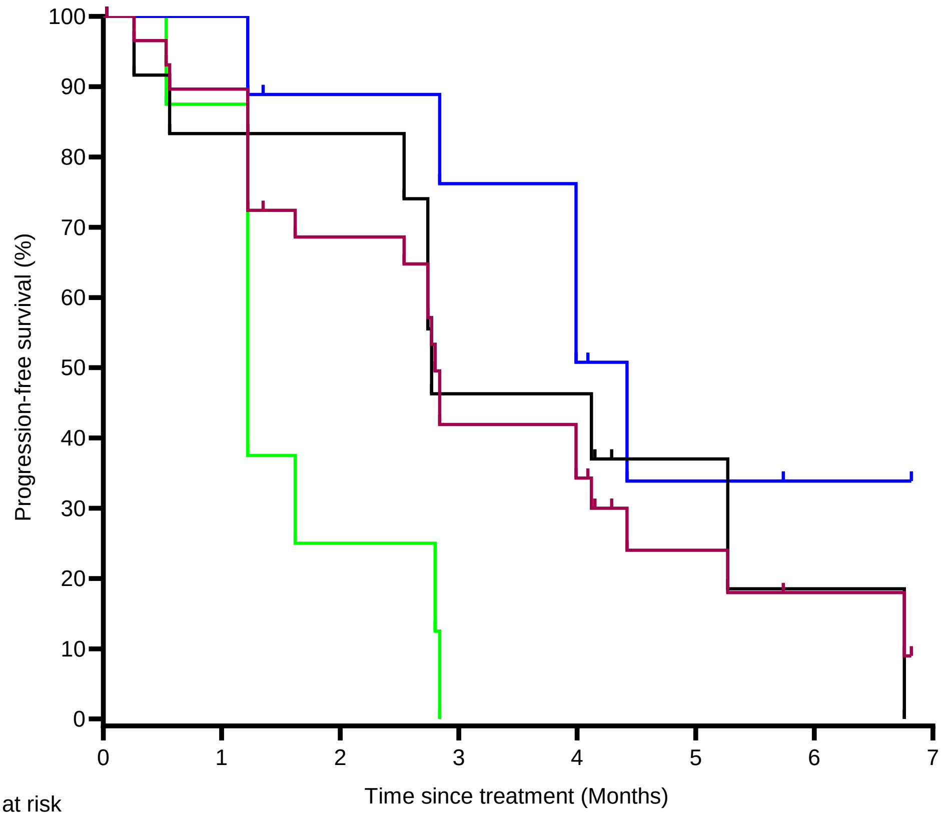

No. at risk								
Total	39	26	18	11	9	4	2	0
SCCHN	13	10	9	5	5	2	1	0
NPC	14	9	7	6	4	2	1	0
CRC	12	7	2	0				

eFigure 2 show the Kaplan-Meier curves of PFS in all Phase Ib patients (n= 39), including squamous cell carcinoma of head neck cancer (SCCHN, n=13), nasopharyngeal cancer (NPC, n=14) and colorectal cancer (CRC, n=12).

© 2022 Qiu M-Z et al. *JAMA Oncology.*
