# Supplementary material for: Evaluation of Safety of Treatment With Anti–Epidermal Growth Factor Receptor Antibody Drug Conjugate MRG003 in Patients With Advanced Solid Tumors: A Phase 1 Nonrandomized Clinical Trial
Source: JAMA Oncol. 2022 May 5;8(7):1042–6. doi: 10.1001/jamaoncol.2022.0503 (PMC9073657; doi:10.1001/jamaoncol.2022.0503)
Supplement: Supplement 3. — Data sharing statement [file jamaoncol-e220503-s003.pdf]

Data Sharing Statement

Qiu. Evaluation of Safety of Treatment With Anti-Epidermal Growth Factor Receptor Antibody Drug Conjugate MRG003 in Patients With Advanced Solid Tumors. *JAMA Oncol*. Published May 05, 2022. doi:10.1001/jamaoncol.2022.0503

Data
Data available: No
